# Endothelin Receptor Antagonism Improves Lipid Profiles and Lowers PCSK9 (Proprotein Convertase Subtilisin/Kexin Type 9) in Patients With Chronic Kidney Disease

**DOI:** 10.1161/HYPERTENSIONAHA.119.12919

**Published:** 2019-07-10

**Authors:** Tariq E. Farrah, Atul Anand, Peter J. Gallacher, Robert Kimmitt, Edwin Carter, James W. Dear, Nicholas L. Mills, David J. Webb, Neeraj Dhaun

**Affiliations:** 1 From the University/British Heart Foundation Centre of Research Excellence, Centre of Cardiovascular Science, University of Edinburgh, Queen’s Medical Research Institute (T.E.F., A.A., P.J.G., R.K., E.C., J.W.D., N.L.M., D.J.W., N.D.); 2 Department of Renal Medicine, Royal Infirmary of Edinburgh (T.E.F., P.J.G., N.D.).

**Keywords:** atherosclerosis, cardiovascular disease, cholesterol, endothelins, triglycerides

## Abstract

Dyslipidemia is common in chronic kidney disease (CKD). Despite statins, many patients fail to adequately lower lipids and remain at increased risk of cardiovascular disease. Selective ET_A_ (endothelin-A) receptor antagonists reduce cardiovascular disease risk factors. Preclinical data suggest that ET_A_ antagonism has beneficial effects on circulating lipids. We assessed the effects of selective ET_A_ antagonism on circulating lipids and PCSK9 (proprotein convertase subtilisin/kexin type 9) in CKD. This was a secondary analysis of a fully randomized, double-blind, 3-phase crossover study. Twenty-seven subjects with predialysis CKD on optimal cardio- and renoprotective treatment were randomly assigned to receive 6 weeks dosing with placebo, the selective ET_A_ receptor antagonist, sitaxentan, or long-acting nifedipine. We measured circulating lipids and PCSK9 at baseline and then after 3 and 6 weeks. Baseline lipids and PCSK9 did not differ before each study phase. Whereas placebo and nifedipine had no effect on lipids, 6 weeks of ET_A_ antagonism significantly reduced total (−11±1%) and low-density lipoprotein–associated (−20±3%) cholesterol, lipoprotein (a) (−16±2%) and triglycerides (−20±4%); high-density lipoprotein–associated cholesterol increased (+14±2%), *P*<0.05 versus baseline for all. Additionally, ET_A_ receptor antagonism, but neither placebo nor nifedipine, reduced circulating PCSK9 (−19±2%; *P*<0.001 versus baseline; *P*<0.05 versus nifedipine and placebo). These effects were independent of statin use and changes in blood pressure or proteinuria. Selective ET_A_ antagonism improves lipid profiles in optimally-managed patients with CKD, effects that may occur through a reduction in circulating PCSK9. ET_A_ receptor antagonism offers a potentially novel strategy to reduce cardiovascular disease risk in CKD.

**Clinical Trial Registration—:**

URL: http://www.clinicaltrials.gov. Unique identifier: NCT00810732.

## Abstract 2

Supplemental Digital Content is available in the text.

Chronic kidney disease (CKD) is common and an important independent risk factor for cardiovascular disease (CVD).^1^ This increased risk is partly explained by a high prevalence of traditional CVD risk factors, such as diabetes mellitus and hypertension.^2^ Dyslipidemia is also common in CKD and contributes to the development of accelerated atherosclerosis and CVD.^3^ HMG-CoA (hydroxymethylglutarate co-enzyme A) reductase inhibitors (statins) lower cholesterol, particularly low-density lipoprotein–associated cholesterol (LDL-C) and have proven efficacy in the reduction of CVD risk in those with and without CKD.^4,5^ However, despite the use of statins, many patients with CKD continue to have elevated lipids.^6^ Furthermore, the side effects associated with these drugs can limit their use.^7^ Thus, novel therapies that might lower cholesterol both in patients established on-statin treatment and in those intolerant of statins would be of major clinical value.

PCSK9 (proprotein convertase subtilisin/kexin type 9) is a serine protease produced mainly in the liver and is an important regulator of tissue LDL-R (LDL receptor) expression and cholesterol homeostasis.^8^ In the circulation, PCSK9 binds to cell surface LDL-R promoting their lysosomal degradation, leading to a rise in circulating LDL-C. Inhibition of circulating PCSK9 using novel humanized monoclonal antibodies leads to important reductions in LDL-C in patients on and off statins.^9,10^ Recent preclinical studies have shown that PCSK9 expression increases during systemic inflammation^11^ and with podocyte injury.^12^ Both are central features of CKD and contributed to by ET-1 (endothelin-1).^13^

ET-1 is the most potent endogenous vasoconstrictor and plays an important role in the development and progression of CKD.^13^ The major pathological effects of ET-1 are mediated via ET_A_ (endothelin-A) receptors.^13^ Preclinical data using ET receptor antagonists have suggested beneficial effects on circulating lipids and atherosclerosis,^14^ but subsequent clinical studies have produced conflicting results, and none have explored potential mechanisms.^15–19^ Thus, we hypothesized that in a cohort of optimally-managed proteinuric patients with CKD, selective ET_A_ receptor antagonism would lead to a reduction in circulating lipids and PCSK9.

## Methods

Data relating to this study are available from the corresponding author on reasonable request. To test the paradigm that selective ET_A_ antagonism can lower circulating lipids, we performed a secondary analysis of a single center, fully randomized, double-blind, 3-phase, and placebo-controlled crossover study in patients with varying degrees of proteinuric, predialysis CKD, a population at significantly increased CVD risk. The full study protocol has been described previously and was performed with subjects’ written consent and South East Scotland research ethics committee approval.^20^

### Patients and Interventions

Twenty-seven patients were enrolled and randomly assigned to receive the selective ET_A_ receptor antagonist, sitaxentan 100 mg once-daily, matched placebo, or long-acting nifedipine 30 mg once-daily for 6 weeks in addition to their usual medications. Each phase was separated by a minimum 2-week washout period. We included patients aged 18 to 70 years of age with stable CKD stages 1 to 4 and proteinuria >300 mg/d. Patients with diabetes mellitus, nephrotic syndrome, significant cardiorespiratory comorbidity, peripheral vascular disease, liver disease, and women of childbearing potential were excluded.

### Assessments

Patients underwent assessments at baseline, week 3 and week 6 of each treatment phase, which included blood sampling for biochemical analyses. Total cholesterol, LDL-C, high-density lipoprotein–associated cholesterol (HDL-C), triglycerides, lipoprotein(a) [Lp(a)], and circulating PCSK9 were also assessed at these timepoints (Figure S1 in the online-only Data Supplement).

### Sample Collection and Analysis

Blood was collected into serum and EDTA tubes, immediately centrifuged at 2500*g* for 20 minutes at 4°C and stored at −80°C until analysis. Lipid parameters were analyzed from stored serum, while PCSK9 was analyzed from stored plasma. Total cholesterol, LDL-C, HDL-C, and triglycerides were measured by enzymatic colorimetric assays. For total cholesterol, the limit of detection and intra-assay and interassay coefficients of variation (CV) were 0.02 mmol/L, 0.8% and 1.3%, respectively. For LDL-C, the limit of detection was 0.03 mmol/L with intra-assay and interassay CV of 1.4% and 2.2%, respectively. For HDL-C, the limit of detection was 0.06 mmol/L with intra-assay and interassay CV of 1.7% and 5.0%, respectively. For triglycerides, the limit of detection was 0.13 mmol/L with intra-assay and interassay CV of 0.8% and 1.7%, respectively. Lp(a) was quantified using a latex agglutination assay with a limit of detection of 0.83 mg/dL and intra-assay and interassay CV of 1.2% and 3.0%, respectively. PCSK9 was measured using an ELISA (R&D systems) with a limit of detection of 0.096 ng/mL. Mean recovery of PCSK9 was 107%, and cross-reactivity was 0% for LDL-R, PCSK1, 3 and 7. The intra-assay and interassay CV were 4.1% and 5.6%, respectively.

### Statistical Analysis

The original study was designed to detect significant changes in proteinuria using data from a prior study,^21^ where an ET_A_ receptor antagonist was administered to 22 subjects in a crossover design leading to a reduction in proteinuria of ≈0.7 g/d with an SD of 0.9 g/d. Using these data, the current study size had 80% power to detect such a difference at the 2-sided 5% significance level.

Baseline lipid levels were assessed by repeated measures 1-way ANOVA with Tukey correction for multiple comparisons to assess carryover and period effect at the start of each treatment phase. A repeated measures 3-way ANOVA was used to assess for interactions between time, treatment, and statin use for changes in lipids and PCSK9. Where Mauchly test indicated the sphericity assumption was not met, the Greenhouse-Geisser or Huynh-Feldt correction were used as appropriate. Changes from baseline to week 6 within treatment phases and between treatments phases at all time points were assessed by repeated measures 2-way ANOVA with Sidak and Tukey corrections for multiple comparisons, respectively. Predictors of change in PCSK9 concentrations were modeled by linear regression, adjusting for potential confounders. Data were analyzed with IBMSPSS (version 24) and R (version 3.3.3). Significance was taken at the 5% level.

## Results

Baseline patient characteristics are shown in Table 1. Baseline mean (±SEM) total cholesterol was 165±29 mg/dL, LDL-C 103±32 mg/dL, HDL-C 43±11 mg/dL mmol/L, triglycerides 143±113 mg/dL, and Lp(a) 31±3 mg/dL. There were no differences in circulating lipids at the start of each study phase (Table 2). Eighteen (67%) patients were prescribed a statin, while 24 (89%) patients were prescribed either an ACEi (angiotensin-converting enzyme inhibitor) or ARB (angiotensin receptor blocker).

**Table 1.**
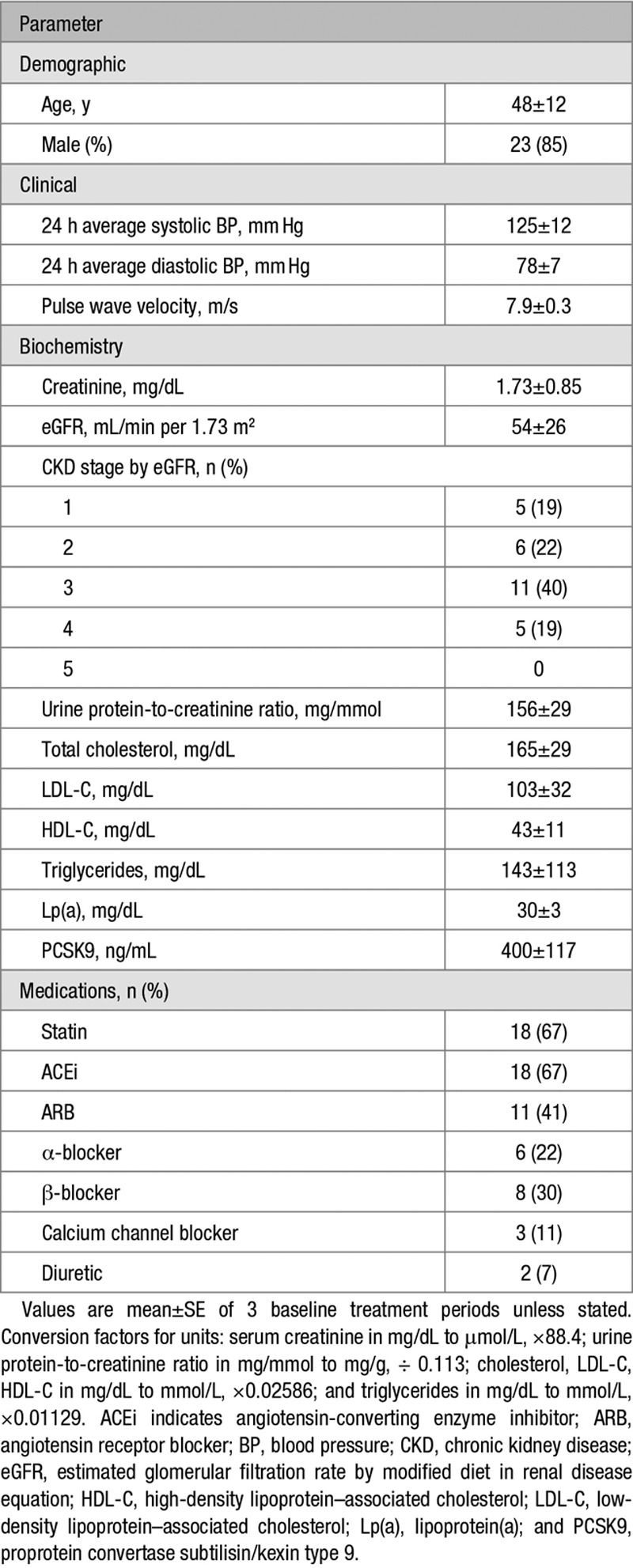
Baseline Study Characteristics

**Table 2.**
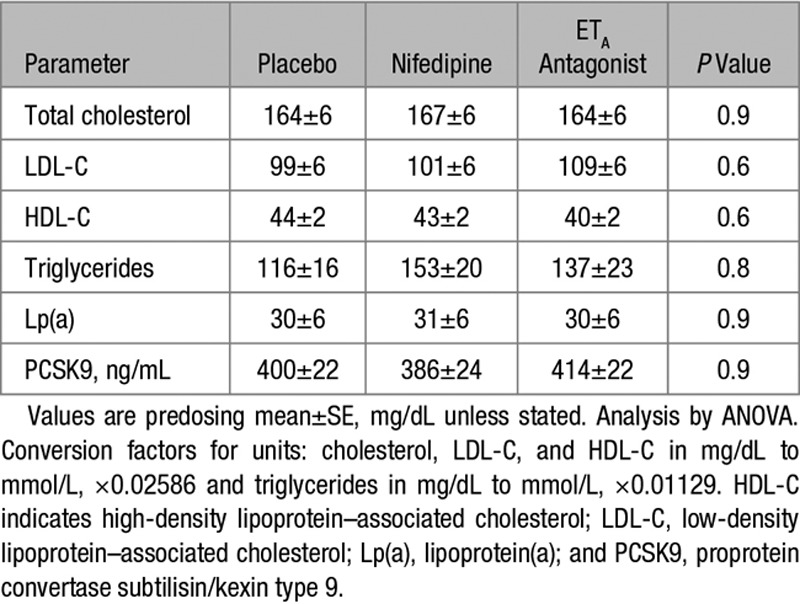
Baseline Lipid Profiles for Each Study Phase

As previously reported,^20^ 6 weeks dosing with a selective ET_A_ antagonist and nifedipine reduced BP and arterial stiffness similarly, but ET_A_ antagonism reduced proteinuria to a greater extent than nifedipine. Placebo did not affect these parameters. All patients completed all 3 phases, and no serious adverse events were recorded during any treatment phase.^20^

**Figure 1.**
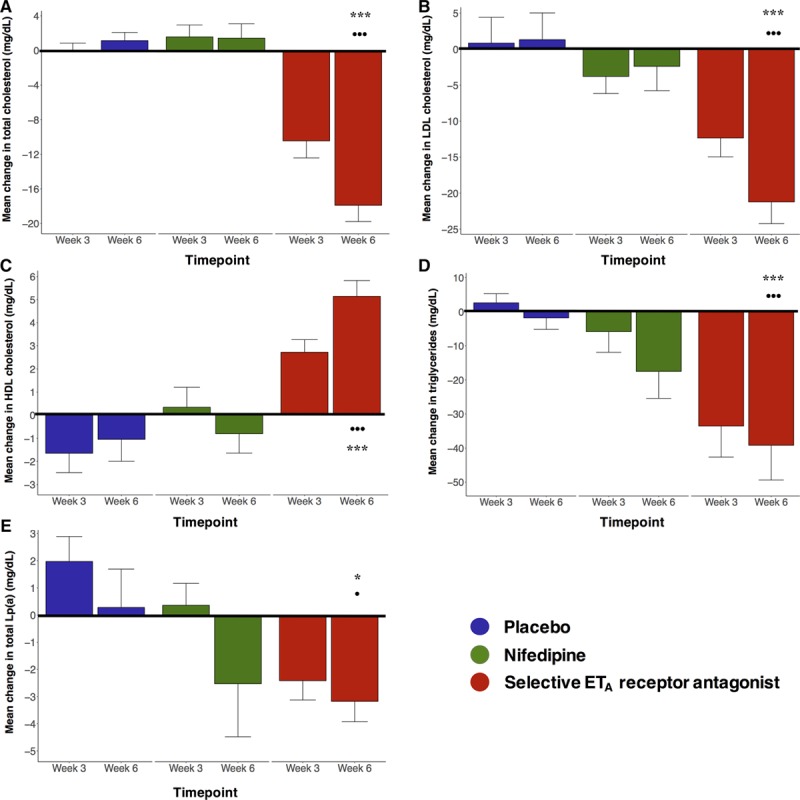
Changes in lipid profiles. Bar chart of mean change from baseline of total cholesterol (**A**), low-density lipoprotein–associated cholesterol (LDL-C); **B**), high-density lipoprotein–associated cholesterol (HDL-C; **C**), triglycerides (**D**), and Lp(a) (lipoprotein(a); **E**) after week 3 and week 6 of dosing with placebo (blue bars), nifedipine (green bars), and selective ET_A_ receptor antagonism (red bars). ***P<0.001 for change at week 6 vs baseline; ^···^*P*<0.001 for change at timepoint vs placebo and nifedipine; and ^·^*P*<0.05 for change at timepoint vs placebo or nifedipine. Analysis by ANOVA. Error bars are SE of mean. Conversion factors for units: cholesterol, LDL-C, and HDL-C in mg/dL to mmol/L, ×0.02586; and triglycerides in mg/dL to mmol/L, ×0.01129.

In terms of effects on circulating lipids and PCSK9, there were no significant 3-way interactions between time, treatment, and statin use but significant interactions between time and treatment were present and analyzed further (Table S1). Total cholesterol, LDL-C, and HDL-C were unaffected by placebo or nifedipine. In contrast, 6 weeks of ET_A_ antagonism led to a fall in total cholesterol of 18±2 mg/dL, a reduction of ≈11% (*P*<0.001 versus baseline; *P*<0.001 versus nifedipine and placebo at week 6, Figure 1A). The reduction in total cholesterol seen with ET_A_ antagonism comprised a fall in LDL-C of 21±3 mg/dL (≈20% reduction, *P*<0.001 versus baseline; *P*<0.001 versus nifedipine and placebo at week 6, Figure 1B) and an increase in HDL-C of 5±1 mg/dL (≈14% increase, *P*<0.001 versus baseline; *P*<0.001 versus nifedipine and placebo at week 6, Figure 1C). ET_A_ antagonism also led to reductions in triglycerides of 39±10 mg/dL (≈20% fall, *P*<0.001 versus baseline; *P*<0.05 versus nifedipine and placebo at week 6, Figure 1D) and in Lp(a) of 3.2±0.8 mg/dL (≈15% fall, *P*<0.05 versus baseline and placebo at week 6, Figure 1E). Detailed effects of ET_A_ antagonism compared with nifedipine and placebo are shown in Tables S2 through S7 with individual patient responses shown in Figures S2 through S6.

**Figure 2.**
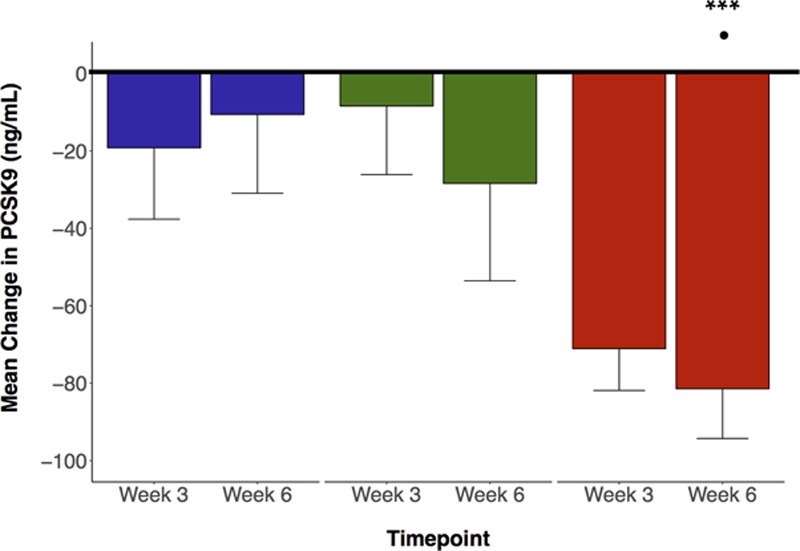
Change in circulating PCSK9 (proprotein convertase subtilisin/kexin type 9). Bar chart of mean change in plasma PCSK9 from baseline after week 3 and week 6 of dosing with placebo (blue bars), nifedipine (green bars), and selective ET_A_ (endothelin-A) receptor antagonist (red bars). ***P<0.001 for selective ET_A_ receptor antagonist at week 6 vs baseline; analysis by paired *t* tests. ^·^*P*<0.05 for change at timepoint vs placebo and nifedipine. Analysis by ANOVA. Error bars are SE of mean.

**Figure 3.**
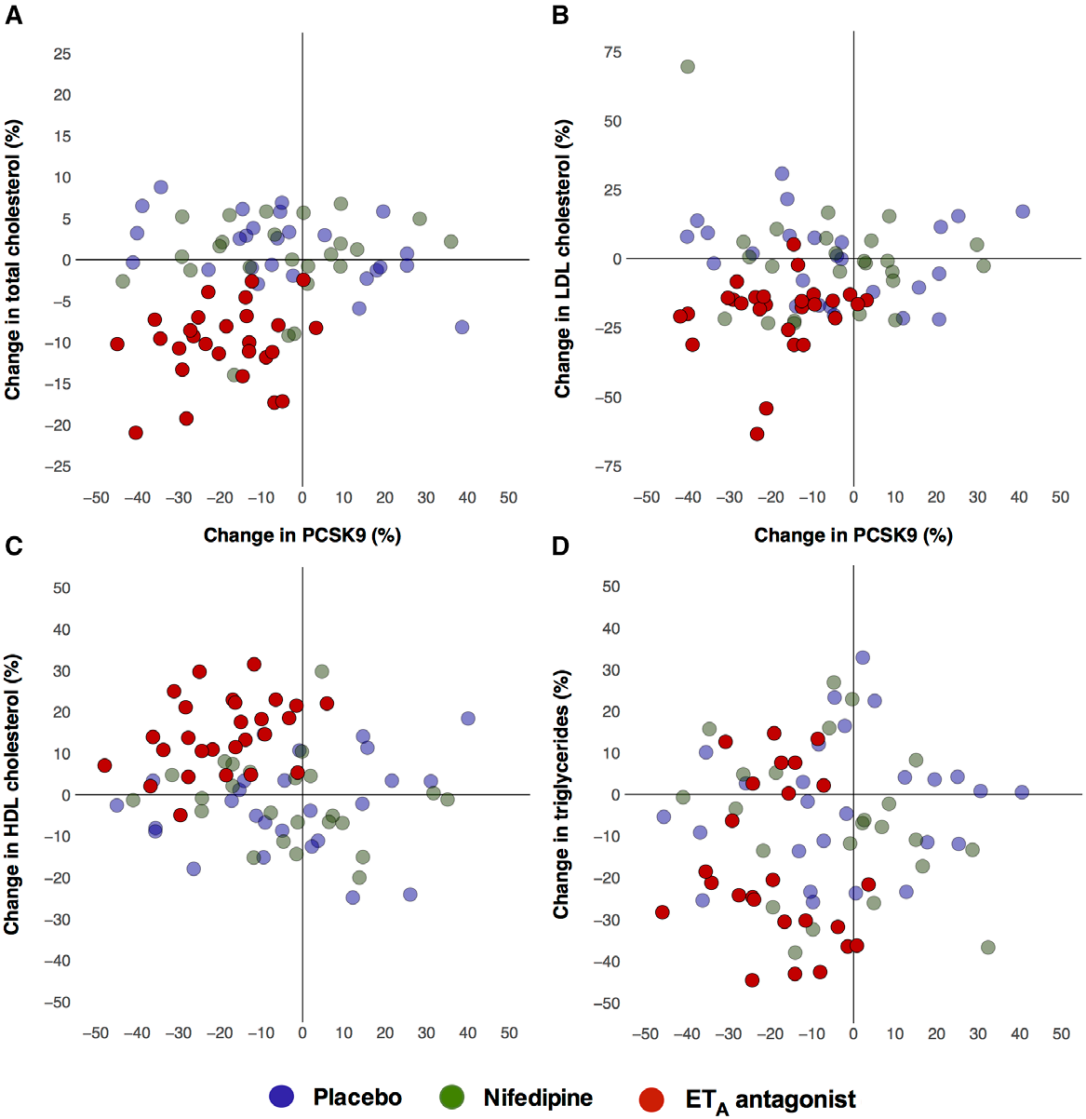
Change in lipids and circulating PCSK9 (proprotein convertase subtilisin/kexin type 9). Scatter plots of individual percentage changes from baseline in total cholesterol (**A**), low-density lipoprotein–associated cholesterol (LDL-C); **B**), high-density lipoprotein–associated cholesterol (HDL-C); **C**), and triglycerides (**D**) after 6 weeks of treatment vs individual percentage change in plasma PCSK9. Blue dots denote subjects receiving placebo; green dots denote subjects receiving nifedipine; and red dots denote subjects receiving selective ET_A_ (endothelin-A) receptor antagonist.

Mean (±SEM) baseline plasma PCSK9 concentration was 400±117 ng/mL and did not differ between the three phases of the study (Table 2). After 6 weeks of ET_A_ receptor antagonism, PCSK9 fell by −81±13 ng/mL (≈20% reduction, *P* <0.001 versus baseline; *P*<0.05 versus nifedipine and placebo, Figure 2 and Figure S7). Reductions in circulating PCSK9 during ET_A_ antagonism were observed to occur with simultaneous reductions in total cholesterol, LDL-C, and to a lesser degree with triglycerides, while also associating with increases in HDL-C (Figure 3). No relationship could be demonstrated for the nifedipine and placebo phases.

Linear regression modeling indicated that treatment with ET_A_ antagonism was an independent predictor of change in PCSK9 concentration at 6 weeks after adjustment for age, sex, statin treatment, and baseline CVD risk factors (adjusted β, 73 ng/mL; 95% CI, −131 to −16; *P*=0.01, Table S8). In addition, selective ET_A_ antagonism remained an independent predictor of change in PCSK9 concentration at 6 weeks after adjustment for change in proteinuria, BP, and pulse wave velocity (Table S9).

## Discussion

Our study has several important findings. We provide new evidence of the broad beneficial effects of selective ET_A_ receptor antagonism on circulating lipids in patients with varying degrees of predialysis CKD and residual proteinuria. Here, medium-term dosing with an ET_A_ antagonist resulted in clinically-relevant reductions in total cholesterol, LDL-C, and triglycerides with a significant increase in HDL-C. In addition, we saw a significant fall in Lp(a). Importantly, these improvements occurred in patients at high CVD risk, the majority of whom were already receiving recommended CVD prevention therapy with a statin and either an ACEi or ARB. Furthermore, we have shown that these lipid-lowering effects occurred with a concurrent reduction in circulating PCSK9. This previously unreported finding supports a link between the endothelin system and cholesterol homeostasis.

Lowering LDL-C has been shown to reduce the risk of major atherosclerotic events in a wide range of patients with CKD.^4^ However, despite current lipid-lowering treatments, a number of patients fail to achieve target LDL-C.^6^ This is mirrored in our cohort of patients whose mean baseline fasting LDL-C was above the recommended 100 mg/dL despite a high rate of statin use.^6,22^ Additionally, of the 18 patients (67%) receiving a statin, 11 were also prescribed the cholesterol-absorption inhibitor, ezetimibe. Thus, from a clinical perspective, it is important that the effects observed with ET_A_ receptor antagonism occurred on top of currently available therapies. Furthermore, suboptimal dosing and discontinuation of statins because of adverse effects remain significant challenges in clinical practice.^7^ For example, the risk of statin-induced myopathy is increased in patients with impaired renal function.^23^ Therefore, alternative agents, which might improve lipid profiles in this particularly high-risk group, while conferring broader CVD risk protection, would be of major clinical value.

Previous reports of the lipid-lowering effects of ET receptor antagonism are limited. Kowala et al^14^ demonstrated that selective ET_A_ antagonism lowered total cholesterol, LDL-C, and triglycerides in hyperlipidemic hamsters, and this reduced aortic arch atherosclerosis. Data from clinical studies are conflicting with some noting an improvement in lipid profiles^15,17^ whereas others have shown no effect.^18,24^ Studies in CKD are limited to those with diabetic nephropathy. In a study using the mixed ET_A/B_ antagonist, avosentan, the authors reported a ≈7% reduction in total cholesterol after 12 weeks dosing but found no effect on triglycerides; they did not report on LDL-C or HDL-C.^16^ Using the selective ET_A_ antagonist, atrasentan, de Zeeuw et al^19^ showed similar effects after 12 weeks on total cholesterol, LDL-C, and triglycerides to those seen in the current study but no change in HDL-C. Our data add to these studies and also demonstrate a beneficial effect on HDL-C and Lp(a), as well as suggesting a potential mechanism through a reduction of circulating PCSK9. Interestingly, these effects on lipids may relate to the relative ET_A_:ET_B_ receptor selectivity of the drug used: bosentan (nonselective) no effect on cholesterol^18^; avosentan (ET_A_:ET_B_, ≈300:1) ≈7% reduction in cholesterol^16^; atrasentan (ET_A_:ET_B_, ≈1200:1) ≈9% reduction in cholesterol^19^; and sitaxentan (ET_A_:ET_B_, ≈6500:1) ≈11% reduction in cholesterol.^25^

Beyond its role in cholesterol homeostasis, a link between circulating PCSK9 concentration and CVD risk has emerged. Leander et al^26^ recently showed in a middle-aged, non-CKD population that a greater circulating PCSK9 was associated with a higher risk of incident CVD, particularly thrombotic events. This remained the case even after adjusting for traditional CVD risk factors including LDL-C and statin use. The mean PCSK9 level in our study (≈400 ng/mL) is comparable to the highest risk quartile identified by Leander et al^26^ and is similar to that seen in other studies in CKD.^27^ The greater CVD risk associated with elevated PCSK9 concentrations may be due in part to its positive association with elevated Lp(a), a modified LDL species that can impair endogenous fibrinolysis.^28^ A recent meta-analysis of >29 000 patients found an independent and near-linear association between both elevated baseline and on-statin Lp(a) concentrations and CVD risk.^29^ This association holds true in CKD as shown in ≈3500 patients with CKD from the Chronic Renal Insufficiency Cohort where a higher concentration of Lp(a) was independently associated with a greater risk of incident myocardial infarction and death during ≈7.5 years follow-up.^30^ Targeting these 2 novel markers of CVD risk using conventional treatments is challenging as statins increase circulating PCSK9^31^ and are ineffective at lowering Lp(a).^32^ Our data suggest that ET_A_ receptor antagonism reduces both.

Inhibition of circulating PCSK9 using novel humanized monoclonal antibodies results in marked reductions in LDL-C, and uniquely Lp(a), in clinical trials of patients both on and off statins.^9,10^ These agents bind to circulating PCSK9, lowering plasma levels by ≈90%.^33^ However, their widespread use is limited by cost; the current list price of evolocumab is more than $14 000 a year per patient.^34^ In our study, ET_A_ receptor antagonism reduced circulating PCSK9 by ≈20%, which was associated with significant improvements in lipids. The pattern of improvement in lipid profiles with ET_A_ receptor antagonism, particularly the significant reductions in Lp(a) (≈16%), is strikingly similar to that observed in clinical trials of PCSK9 inhibitors^9,10^ (Figure S8) suggesting that these are secondary to a reduction in circulating PCSK9.

**Figure 4.**
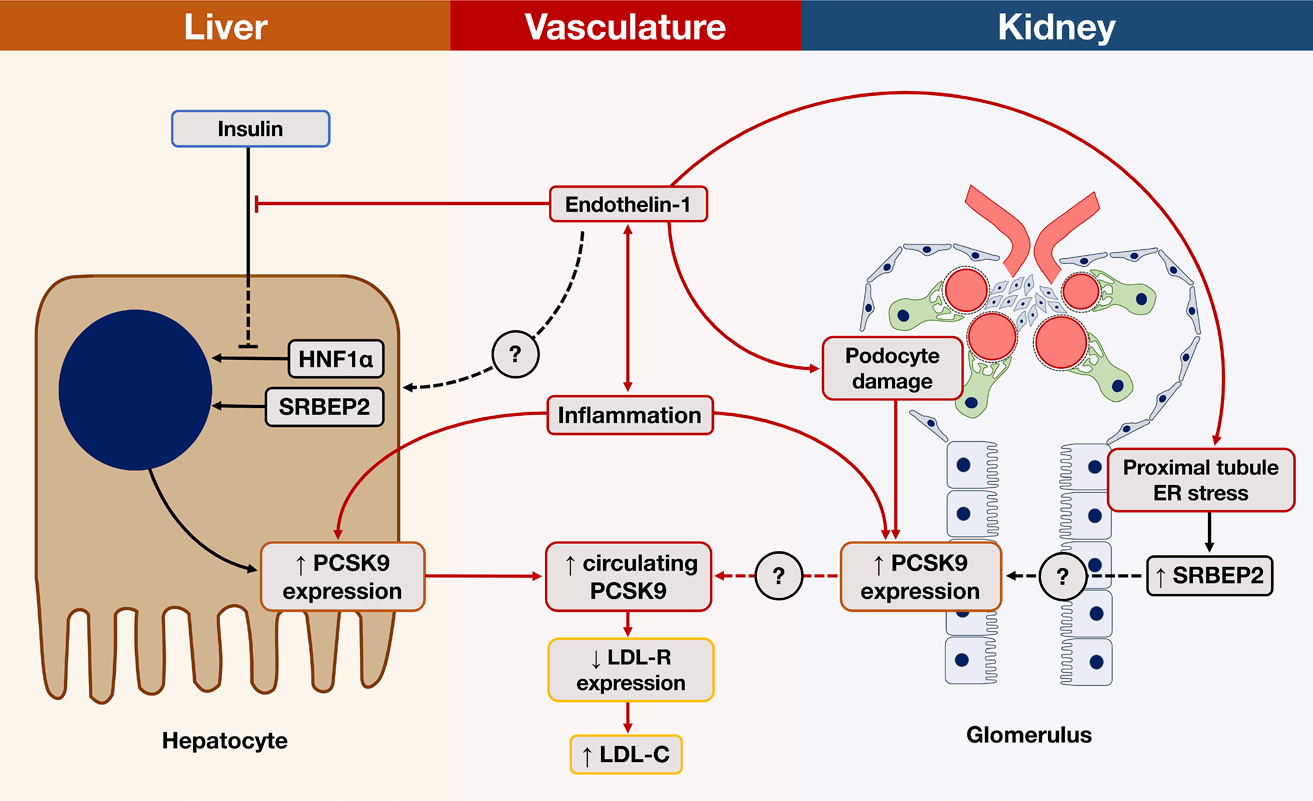
Proposed pathways linking the endothelin system, PCSK9 (proprotein convertase subtilisin/kexin type 9) expression and cholesterol in chronic kidney disease. The liver is the major site of PCSK9 expression with HNF1α (hepatic nuclear factor 1α) and SREBP2 (sterol regulatory element binding protein 2) its principle promoters.^4^ Animals studies have shown that insulin binding to hepatocytes prevents nuclear translocation of HNF1α thus reducing PCSK9 transcription.^5^ ET-1 (endothelin-1) impairs hepatocyte insulin sensitivity^6^ which can be ameliorated by selective ET_A_ receptor antagonism^7,8^ and so may restore the inhibitory effect of insulin on HNF1α-mediated PCSK9 transcription in hepatocytes. Whether ET-1 has direct effects on HNF1α or SREBP2 in hepatocytes is unknown. Systemic inflammation can increase both hepatic and renal PCSK9 expression with a concurrent reduction in LDL-R (low density lipoprotein receptor) expression^9^ and a rise in low-density lipoprotein–associated cholesterol (LDL-C). In the vasculature, ET-1 has proinflammatory effects mediated predominantly through ET_A_ receptor activation.^10^ In the kidney, podocyte damage is associated with increased circulating and renal PCSK9 expression, notably localized to proximal tubular cells in murine models.^11^ The relevance of renal PCSK9 expression to the circulating PCSK9 pool and lipids needs further clarification. Interestingly, ER (endoplasmic reticulum) stress leads to an upregulation of SREBP2 in renal proximal tubular cells with subsequent apoptosis,^12^ but effects on PCSK9 expression here are unexplored. However, selective ET_A_ antagonism has been shown to ameliorate podocyte injury^13^ and proximal tubule ER stress^14^ suggesting a potential role in renal PCSK9 expression.

Our observed reduction in PCSK9 during ET_A_ antagonism occurred independently of the major recognized vascular and renal effects of this drug class, namely reductions in BP, arterial stiffness, and proteinuria. This suggests novel and specific links between the endothelin system and PCSK9, as proposed in Figure 4. Systemic inflammation, podocyte injury, and proteinuria are features of CKD and in which ET_A_ receptor activation plays a key role.^13^ All 3 have also been shown to increase tissue and circulating PCSK9 expression in mice.^11,12^ However, there was inconsistent evidence in these studies of a concurrent upregulation of SREBP2 (sterol regulatory element binding protein 2) and HNF1α (hepatic nuclear factor 1α), the principle promoters of PCSK9 expression. Induction of endoplasmic reticulum stress in murine renal proximal tubular cells has been shown to increase SREBP2 expression with subsequent lipid accumulation and apoptosis.^35^ In a separate study, using the same model, selective ET_A_ antagonism reduced endoplasmic reticulum stress and apoptosis of proximal tubular cells.^36^ These studies did not examine PCSK9 expression, but recent work suggests that increased renal PCSK9 expression after podocyte injury/ablation is also localized to proximal tubular cells.^12^

Other preclinical data suggest an important role for insulin in reducing hepatic PCSK9 expression by preventing nuclear translocation of HNF1α.^37^ ET-1 has been to shown to promote hepatic insulin resistance^38^ which can be restored by ET_A_ receptor antagonism in Zucker fatty rats^39^ and in man^40^ with a subsequent improvement in glucose metabolism. Finally, inflammation may act as an important shared pathway as vascular smooth muscle cells, the predominant site of ET_A_ receptor expression, have recently been shown to express PCSK9^41^ which can be upregulated by inflammatory stimuli in vitro.^42^

The collected published data link the endothelin system, PCSK9 and lipid homeostasis (Figure 4) and provide a plausible mechanistic basis for our clinical observations that should be explored further in future studies. We recognize the small size of our study as well as its medium-term duration and observational nature and while our data are secondary analyses, they originate from a well-designed, fully randomized, placebo-controlled clinical trial with no measurable evidence of carryover or period effects. We acknowledge that while the 2-week washout period between phases was designed to ensure adequate drug elimination, persisting effects on cholesterol metabolism cannot be fully excluded, although our detailed analyses show no sign of this. We demonstrate clear, consistent benefits on circulating lipids with ET_A_ antagonism and provide novel insight into links between the endothelin system and cholesterol homeostasis in kidney disease.

## Perspectives

Medium-term selective ET_A_ antagonism improves lipid profiles in optimally-managed patients with CKD. Our data suggest that the lipid-lowering effects of ET_A_ antagonism may be achieved through a reduction in circulating PCSK9. Alongside recognized reductions in BP, proteinuria and arterial stiffness, ET_A_ receptor antagonism offers a novel strategy to reduce CVD risk in patients with CKD. Current larger clinical trials of selective ET_A_ antagonism alone^43^ or in combination with angiotensin II receptor blockade^43,44^ in patients with proteinuric CKD should help confirm the current observations.

## Acknowledgments

Drs Farrah and Anand drafted the article, performed statistical analysis, and critically revised the article. Dr Gallacher performed statistical analysis and critically revised the article. Dr Kimmitt critically revised the article. E. Carter performed biochemical assays. Drs Dear, Mills, and Webb critically revised the article. Dr Dhaun conceived the study, carried out the primary study, and critically revised the article. All authors approved the final version.

## Sources of Funding

Dr Farrah is supported by a Clinical Research Training Fellowship from the Medical Research Council (MR/R017840/1). Dr Anand is supported by a Research Fellowship from Chest Heart and Stroke Scotland (15/A163). Dr Mills is supported by the Butler Senior Research Fellowship (FS/16/14/32023) and Chair (CH/09/002) awards from the British Heart Foundation. Dr Dhaun is supported by a BHF Intermediate Clinical Research Fellowship (FS/13/30/29994). The original study was funded by Pfizer Inc.

## Disclosures

Dr Dhaun has acted as a consultant for Retrophin Inc. The other authors report no conflicts.

## Supplementary Material

## Novelty and Significance

### What Is New?

- In patients with chronic kidney disease, treatment with a selective ET_A_ (endothelin-A) antagonist led to clinically-relevant reductions in circulating lipids.
- Interestingly, these lipid-lowering effects occurred alongside a reduction in circulating PCSK9 (proprotein convertase subtilisin/kexin type 9).

### What Is Relevant?

- Patients with chronic kidney disease are at high cardiovascular risk. Dyslipidemia is common and despite the use of statins, many of these patients continue to have elevated lipids.
- The beneficial effects seen here were in patients at high cardiovascular risk, the majority of whom were already receiving recommended cardiovascular disease prevention therapy.

### Summary

In patients with predialysis chronic kidney disease, selective ET_A_ receptor antagonism reduces circulating lipids. These effects are seen on top of statin therapy and may be mediated through a reduction in PCSK9. These previously unreported findings support a link between the endothelin system and cholesterol homeostasis.
