# Supplementary material for: Endothelin Receptor Antagonism Improves Lipid Profiles and Lowers PCSK9 (Proprotein Convertase Subtilisin/Kexin Type 9) in Patients With Chronic Kidney Disease
Source: Hypertension. 2019 Jul 10;74(2):323–30. doi: 10.1161/HYPERTENSIONAHA.119.12919 (PMC6635059; doi:10.1161/HYPERTENSIONAHA.119.12919)

SUPPLEMENTARY APPENDIX

**Endothelin receptor antagonism improves lipid profiles & lowers PCSK9 in patients with chronic kidney disease**

Tariq E Farrah^* 1, 2^

Atul Anand^* 1^

Peter J Gallacher^1,2^

Robert Kimmitt^1^

Edwin Carter^1^

James W Dear^1^

Nicholas L Mills^1^

David J Webb^1^

Neeraj Dhaun^1, 2^ ** Joint first authors*

^1^ University/British Heart Foundation Centre of Research Excellence, Centre of Cardiovascular Science, University of Edinburgh, Queen's Medical Research Institute, 47 Little France Crescent, Edinburgh, EH16 4TJ

^2^ Department of Renal Medicine, Royal Infirmary of Edinburgh, 51 Little France Crescent, Edinburgh, EH16 4SA

**Running title**: Endothelin & lipids in chronic kidney disease

**Correspondence to**: Dr Neeraj Dhaun (*Bean)*

Centre for Cardiovascular Science

The Queen’s Medical Research Institute

47 Little France Crescent, Edinburgh.

EH16 4TJ

[bean.dhaun@ed.ac.uk](mailto:bean.dhaun@ed.ac.uk)

Pages: 15

**Contents**

**1. References**

**2. Supplementary data table S1.** *3-way ANOVA*

**3. Supplementary data tables S2-S7**. *Data tables*

**4**. **Supplementary data table S8.** *Linear regression modeling of predictors of change in PCSK9 by baseline parameters*

**5. Supplementary data table S9.** *Linear regression modeling of predictors of change in PCSK9 by change in primary endpoints*

**6. Supplementary figure S1.** *Study overview*

**7. Supplementary figures S2-S6.** *Individual patient changes in lipids for each treatment phase*

**8**. **Supplementary figure S7.** *Individual patient changes in PCSK9 for each treatment phase*

**9. Supplementary figure S8.** *Pattern of change in lipid profile with ET_A_ receptor antagonist compared to PCSK9 inhibitors*

**1.**  **References**

1. Dhaun N, MacIntyre IM, Kerr D, Melville V, Johnston NR, Haughie S, Goddard J, Webb DJ. Selective endothelin-A receptor antagonism reduces proteinuria, blood pressure, and arterial stiffness in chronic proteinuric kidney disease. Hypertension 2011;**57**:772-9.

2. Sabatine MS, Giugliano RP, Wiviott SD, Raal FJ, Blom DJ, Robinson J, Ballantyne CM, Somaratne R, Legg J, Wasserman SM, Scott R, Koren MJ, Stein EA, Open-Label Study of Long-Term Evaluation against LDLCI. Efficacy and safety of evolocumab in reducing lipids and cardiovascular events. N Engl J Med 2015;**372**:1500-9.

3. Robinson JG, Farnier M, Krempf M, Bergeron J, Luc G, Averna M, Stroes ES, Langslet G, Raal FJ, El Shahawy M, Koren MJ, Lepor NE, Lorenzato C, Pordy R, Chaudhari U, Kastelein JJ, Investigators OLT. Efficacy and safety of alirocumab in reducing lipids and cardiovascular events. N Engl J Med 2015;**372**:1489-99.

**1. Supplementary data table S1**. Repeated measures 3-way ANOVA to assess interactions between time, treatment and concomitant statin use for changes in lipids and PCSK9. Where Mauchly’s test indicated the sphericity assumption was not met, the Greenhouse-Geisser or Huynh-Feldt correction were used as appropriate. Significance taken at 5% level. LDL-C – low-density lipoprotein; HDL-C– high-density lipoprotein Lp(a) – lipoprotein (a); PCSK9 – proprotein convertase subtilisin/kexin type 9; Sig. – significance; n/a – not applicable.

| **Time** |  | | | | | | | |
| --- | --- | --- | --- | --- | --- | --- | --- | --- |
| **Parameter** | **Levene's equality**  **of error variances** | | **Mauchly's test**  **for sphericity** | **Sig.** | **Greenhouse-**  **Geisser ε** | **F** | **dF** | **p value** |
| Cholesterol | 0.752 | | 0.890 | 0.013 | 0.901 | 17.813 | 1.966 | **<0.001** |
| LDL-C | 0.632 | | 0.762 | <0.001 | 0.808 | 7.220 | 1.756 | **0.002** |
| HDL-C | 0.733 | | 0.991 | 0.708 | n/a | 3.347 | 2.000 | **0.038** |
| Triglycerides | 0.648 | | 0.365 | <0.001 | 0.612 | 13.315 | 1.223 | **<0.001** |
| Lp(a) | 0.518 | | 0.650 | <0.001 | 0.741 | 3.450 | 1.481 | **0.049** |
| PCSK9 | 0.706 | | 0.981 | 0.498 | n/a | 7.714 | 2.000 | **0.001** |
|  |  | |  |  |  |  |  |  |
| **Time * treatment** | |  |  |  |  |  |  |  |
| **Parameter** | **Levene's equality**  **of error variances** | | **Mauchly's test**  **for sphericity** | **Sig.** | **Greenhouse-**  **Geisser ε** | **F** | **dF** | **p value** |
| Cholesterol | 0.752 | | 0.890 | 0.013 | 0.901 | 29.244 | 3.932 | **<0.001** |
| LDL | 0.632 | | 0.762 | <0.001 | 0.808 | 6.900 | 3.513 | **<0.001** |
| HDL | 0.733 | | 0.991 | 0.708 | n/a | 9.491 | 4.000 | **<0.001** |
| Triglycerides | 0.648 | | 0.365 | <0.001 | 0.612 | 6.791 | 2.446 | **0.001** |
| Lp(a) | 0.518 | | 0.650 | <0.001 | 0.741 | 2.716 | 2.962 | **0.049** |
| PCSK9 | 0.706 | | 0.981 | 0.498 | n/a | 3.174 | 4.000 | **0.016** |
|  |  | |  |  |  |  |  |  |
| **Time * statin use** | |  |  |  |  |  |  |  |
| **Parameter** | **Levene's equality**  **of error variances** | | **Mauchly's test**  **for sphericity** | **Sig.** | **Greenhouse-**  **Geisser ε** | **F** | **dF** | **p value** |
| Cholesterol | 0.752 | | 0.890 | 0.013 | 0.901 | 0.526 | 1.966 | 0.589 |
| LDL | 0.632 | | 0.762 | <0.001 | 0.808 | 1.552 | 1.756 | 0.217 |
| HDL | 0.733 | | 0.991 | 0.708 | n/a | 0.703 | 2.000 | 0.497 |
| Triglycerides | 0.648 | | 0.365 | <0.001 | 0.612 | 0.340 | 1.223 | 0.897 |
| Lp(a) | 0.518 | | 0.650 | <0.001 | 0.741 | 0.767 | 1.481 | 0.431 |
| PCSK9 | 0.706 | | 0.981 | 0.498 | n/a | 0.092 | 2.000 | 0.909 |
|  | | |  |  |  |  |  |  |
| **Time * treatment * statin** | | |  |  |  |  |  |  |
| **Parameter** | **Levene's equality**  **of error variances** | | **Mauchly's test**  **for sphericity** | **Sig.** | **Greenhouse-**  **Geisser ε** | **F** | **dF** | **p value** |
| Cholesterol | 0.752 | | 0.890 | 0.013 | 0.901 | 0.281 | 3.932 | 0.887 |
| LDL | 0.632 | | 0.762 | <0.001 | 0.808 | 1.414 | 3.513 | 0.237 |
| HDL | 0.733 | | 0.991 | 0.708 | n/a | 0.633 | 4.000 | 0.640 |
| Triglycerides | 0.648 | | 0.365 | <0.001 | 0.612 | 0.709 | 2.446 | 0.521 |
| Lp(a) | 0.518 | | 0.650 | <0.001 | 0.741 | 1.200 | 2.962 | 0.313 |
| PCSK9 | 0.706 | | 0.981 | 0.498 | n/a | 1.379 | 4.000 | 0.244 |

**2.** **Supplementary data tables S2 – S7**. Values are mean absolute change ± SEM. Within phase comparisons are by repeated measures 2-way ANOVA with Sidak’s multiple comparisons test between baseline and week 6. Between phase comparisons are by 2-way ANOVA with Tukey correction for multiple comparisons. Significance taken at 5% level.

Supplementary table S2. *Total cholesterol (mg/dL)*

|  | Change from baseline at week 3 | Change from baseline at week 6 | *p value of*  *change from baseline at week 6* |
| --- | --- | --- | --- |
| Placebo | 0.0 ± 0.9 | 1.1 ± 0.9 | 0.2 |
| Nifedipine | 1.6 ± 1.4 | 1.4 ± 1.7 | 0.4 |
| Sitaxentan | -10.5 ± 2.0 | -17.9 ± 1.9 | **<0.001** |
| *p value nifedipine vs. placebo* | 0.46 | 0.89 |  |
| *p value sitaxentan vs. placebo* | **<0.001** | **<0.001** |  |
| *p value sitaxentan vs. nifedipine* | **<0.001** | **<0.001** |  |

Supplementary table S3. *LDL-C (mg/dL)*

|  | Change from baseline at week 3 | Change from baseline at week 6 | *p value*  *change from baseline at week 6* |
| --- | --- | --- | --- |
| Placebo | 0.8 ± 3.6 | 1.3 ± 3.7 | 0.4 |
| Nifedipine | -3.8 ± 2.4 | -2.4 ± 3.4 | 0.6 |
| Sitaxentan | -12.3 ± 2.6 | -21.2 ± 3.0 | **<0.001** |
| *p value nifedipine vs. placebo* | 0.30 | 0.41 |  |
| *p value sitaxentan vs. placebo* | **0.004** | **<0.001** |  |
| *p value sitaxentan vs. nifedipine* | 0.06 | **<0.001** |  |

LDL-C – low-density lipoprotein cholesterol.

Supplementary table S4. *HDL-C (mg/dL)*

|  | Change from baseline at week 3 | Change from baseline at week 6 | *p value of*  *change from baseline at week 6* |
| --- | --- | --- | --- |
| Placebo | -1.6 ± 0.8 | -1.0 ± 1.0 | 0.3 |
| Nifedipine | 0.3 ± 0.9 | -0.8 ± 0.8 | 0.5 |
| Sitaxentan | 2.7 ± 0.6 | 5.1 ± 0.7 | **<0.001** |
| *p value nifedipine vs. placebo* | 0.1 | 0.9 |  |
| *p value sitaxentan vs. placebo* | **<0.001** | **<0.001** |  |
| *p value sitaxentan vs. nifedipine* | **0.038** | **<0.001** |  |

HDL-C – high-density lipoprotein cholesterol. Conversion factor for HDL-C in mg/dL to mmol/L, x 0.02586

Supplementary table S5. *Triglycerides (mg/dL)*

|  | Change from baseline at week 3 | Change from baseline at week 6 | *p value of*  *change from baseline at week 6* |
| --- | --- | --- | --- |
| Placebo | 2.6 ± 2.7 | -1.8 ± 3.4 | 0.5 |
| Nifedipine | -5.8 ± 6.1 | -17.5 ± 7.9 | 0.06 |
| Sitaxentan | -33.5 ± 9.1 | -39.2 ± 10.2 | **<0.001** |
| *p value nifedipine vs. placebo* | 0.4 | 0.1 |  |
| *p value sitaxentan vs. placebo* | **<0.001** | **<0.001** |  |
| *p value sitaxentan vs. nifedipine* | **0.007** | **0.033** |  |

Conversion factor for Triglycerides in mg/dL to mmol/L, x 0.01129

Supplementary table S6. *Lp(a) (mg/dL)*

|  | Change from baseline at week 3 | Change from baseline at week 6 | *p value of*  *change from baseline at week 6* |
| --- | --- | --- | --- |
| Placebo | 2.0 ± 0.9 | 0.3 ± 1.4 | 0.4 |
| Nifedipine | 0.4 ± 0.8 | -2.5 ± 2.0 | 0.2 |
| Sitaxentan | -2.4 ± 0.7 | -3.2 ± 0.8 | **0.04** |
| *p value nifedipine vs. placebo* | 0.3 | 0.1 |  |
| *p value sitaxentan vs. placebo* | **0.009** | **0.04** |  |
| *p value sitaxentan vs. nifedipine* | 0.099 | 0.65 |  |

Lp(a) – lipoprotein a

Supplementary table S7. *PCSK9 (ng/mL)*

|  | Change from baseline at week 3 | Change from baseline at week 6 | *p value of*  *change from baseline at week 6* |
| --- | --- | --- | --- |
| Placebo | -19.2 ± 18.4 | -10.6 ± 20.4 | 0.6 |
| Nifedipine | -8.4 ± 17.7 | -28.4 ± 25.2 | 0.6 |
| Sitaxentan | -71.1 ± 10.8 | -81.4 ± 12.9 | **<0.001** |
| *p value nifedipine vs. placebo* | 0.7 | 0.5 |  |
| *p value sitaxentan vs. placebo* | **0.045** | **0.007** |  |
| *p value sitaxentan vs. nifedipine* | **0.016** | **0.041** |  |

PCSK9 – proprotein convertase subtilisin/kexin 9

**3. Supplementary data table S8.** *Linear regression modeling of predictors of change in PCSK9 by baseline parameters.* Values are beta-coefficients for the change in PCSK9 per unit change in variable listed (95% confidence interval) **p<0.05*

| **Variable** | **Model 1 β** | **Model 2 β** | **Model 3 β** | **Model 4 β** | **Model 5 β** |
| --- | --- | --- | --- | --- | --- |
| Age, per year | 1.5  (-0.6 – 3.5) | 1.5  (-0.6 − 3.5) | 1.5  (-0.6 − 3.5) | 1.5  (-0.6 − 3.6) | 0.3  (-2.4 − 3.1) |
| Male sex | 21.2  (-44.4 – 86.9) | 12.0  (-53.2 –77.1) | 12.0  (-53.7 –77.6) | 11.8  (-58.9 − 82.6) | 1.0  (-71.3 − 73.4) |
| Statin therapy | 9.0  (-41.8 – 59.9) | 17.8  (-33.0 – 68.5) | 17.7  (-33.5 – 68.9) | 17.7  (-34.0 – 69.5) | 13.7  (-38.2 – 65.6) |
| Nifedipine  (reference placebo) | -17.8  (-74.7 – 39.1) | -18.3  (-74.2 – 37.6) | -18.3  (-74.6 – 38.0) | -18.3  (-75.0 – 38.4) | -21.3  (-77.9 – 35.4) |
| Selective ET_A_ antagonist  (reference placebo) | **-70.9***  (-127.8 – -14.0) | **-69.0***  (-124.9 − -13.0) | **-68.7***  (-125.5 − -11.8) | **-68.7***  (-126.0 − -11.3) | **-73.1***  (-130.6 – -15.6) |
| Baseline systolic BP, per mmHg |  | 1.8  (-0.1 – 3.7) | 1.8  (-0.1 – 3.8) | 1.8  (-0.3 – 4.0) | 1.8  (-0.3 – 3.9) |
| Baseline LDL-C,  per mg/dL |  |  | 0.0  (-0.8 – 0.7) | 0.0  (-0.8 – 0.8) | 0.1  (-0.7 – 0.9) |
| Baseline urine PCR, per mg/mmol |  |  |  | 0.0  (-0.2 – 0.2) | 0.0  (-0.2 – 0.2) |
| Baseline PWV,  per m/s |  |  |  |  | 13.1  (-7.0 – 33.1) |

PCSK9 – proprotein convertase subtilisin/kexin type 9; ET_A_ – endothelin-A; BP – blood pressure; LDL-C – low density lipoprotein cholesterol; PCR – protein-to-creatinine ratio; PWV – pulse wave velocity

**4.** **Supplementary data table S9.** *Linear regression modeling of predictors of change in PCSK9 by change in primary endpoints.* Figures are beta-coefficients for the change in PCSK9 per unit change in variable listed (95% confidence interval). *p<0.05, **p<0.01

| **Variable** | **Model 1 β** | **Model 2 β** | **Model 3 β** | **Model 4 β** |
| --- | --- | --- | --- | --- |
| Nifedipine  (reference placebo) | -17.8  (-74.4 – 38.8) | -17.9  (-74.6 – 38.8) | -24.5  (-82.2 – 33.2) | -11.6  (-70.9 – 47.8) |
| Selective ET_A_ receptor anatagonist  (reference placebo) | -70.9*  (-127.5 – -14.2) | -79.2**  (-138.4 − -20.1) | -75.2*  (-132.3 – -18.2) | -64.4*  (-123.9 – -4.8) |
| Change in PCR,  per mg/mmol |  | -0.2  (-0.6 – 0.2) |  |  |
| Change in systolic BP, per mmHg |  |  | -1.8  (-4.8 – 1.3) |  |
| Change in PWV,  per m/s |  |  |  | 9.2  (-16.3 – 34.7) |

PCSK9 – proprotein convertase subtilisin/kexin type 9; ET_A_ – endothelin-A; BP – blood pressure; PCR – urine protein to creatinine ratio; PWV – pulse wave velocity

**1. Supplementary figure S1.** *Study overview^1^*

CKD – chronic kidney disease; g/d – grams per day; CVD – cardiovascular disease; ACEi – angiotensin converting enzyme inhibitor; ARB – angiotensin receptor blocker; ET_A_ – endothelin-A receptor; LDL-C – low-density lipoprotein cholesterol, HDL-C – high-density lipoprotein cholesterol; PCSK9 – proprotein convertase subtilisin/kexin type 9; BP – blood pressure.

**6. Supplementary figures S2-S6.** *Individual patient changes in lipids and PCSK9 for each treatment phase. ‘*Before and after’ plots of individual patient lipid concentrations at baseline and week 6 for each treatment phase. Blue dots denote subjects on placebo; green dots denote subjects on nifedipine, red dots denote subject on selective ET_A_ receptor antagonist. LDL-C – low-density lipoprotein; HDL-C– high-density lipoprotein; ET_A_ – endothelin-A; PCSK9 – proprotein convertase subtilisin/kexin type 9.

Supplementary figure S2. *Total cholesterol*


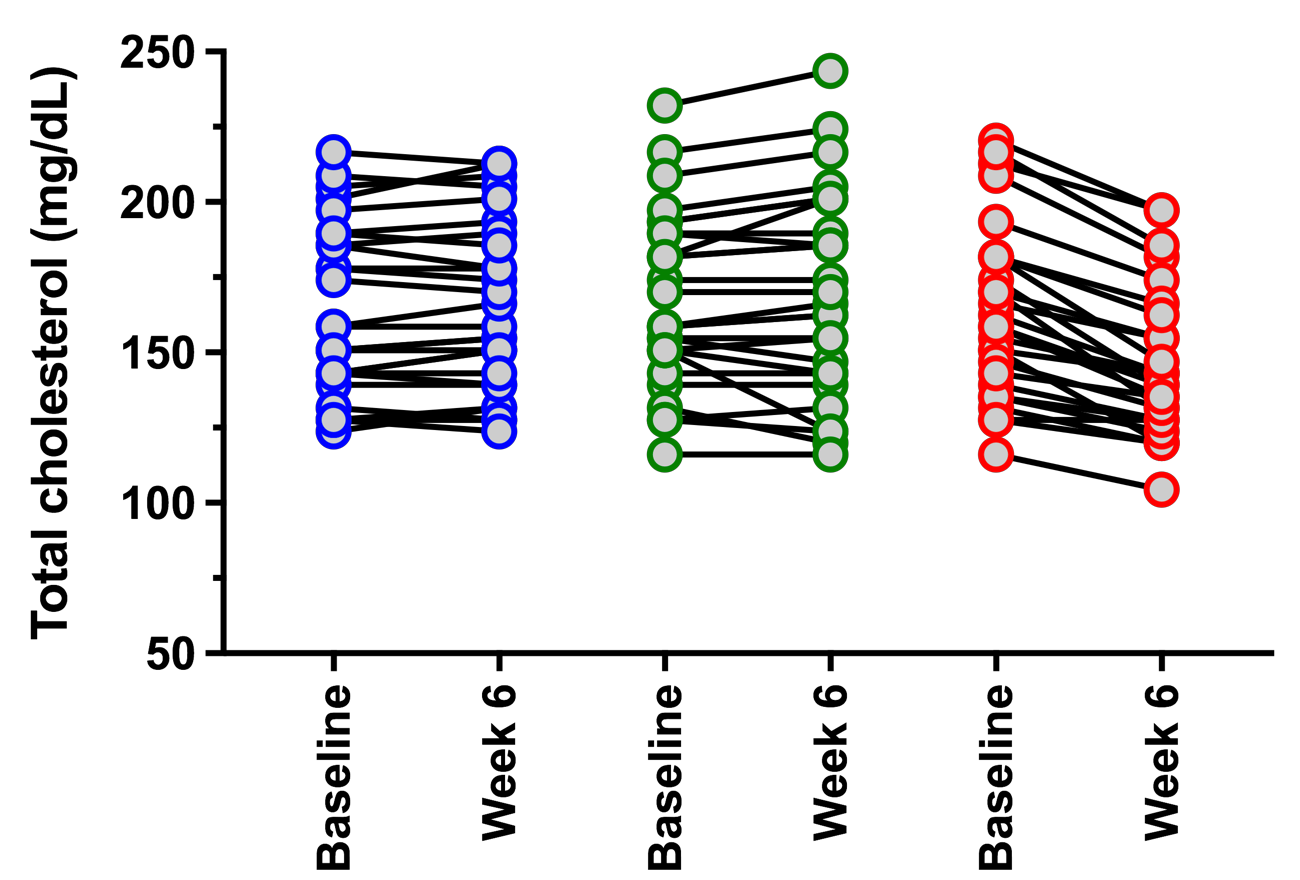


Supplementary figure S3*. LDL-cholesterol*

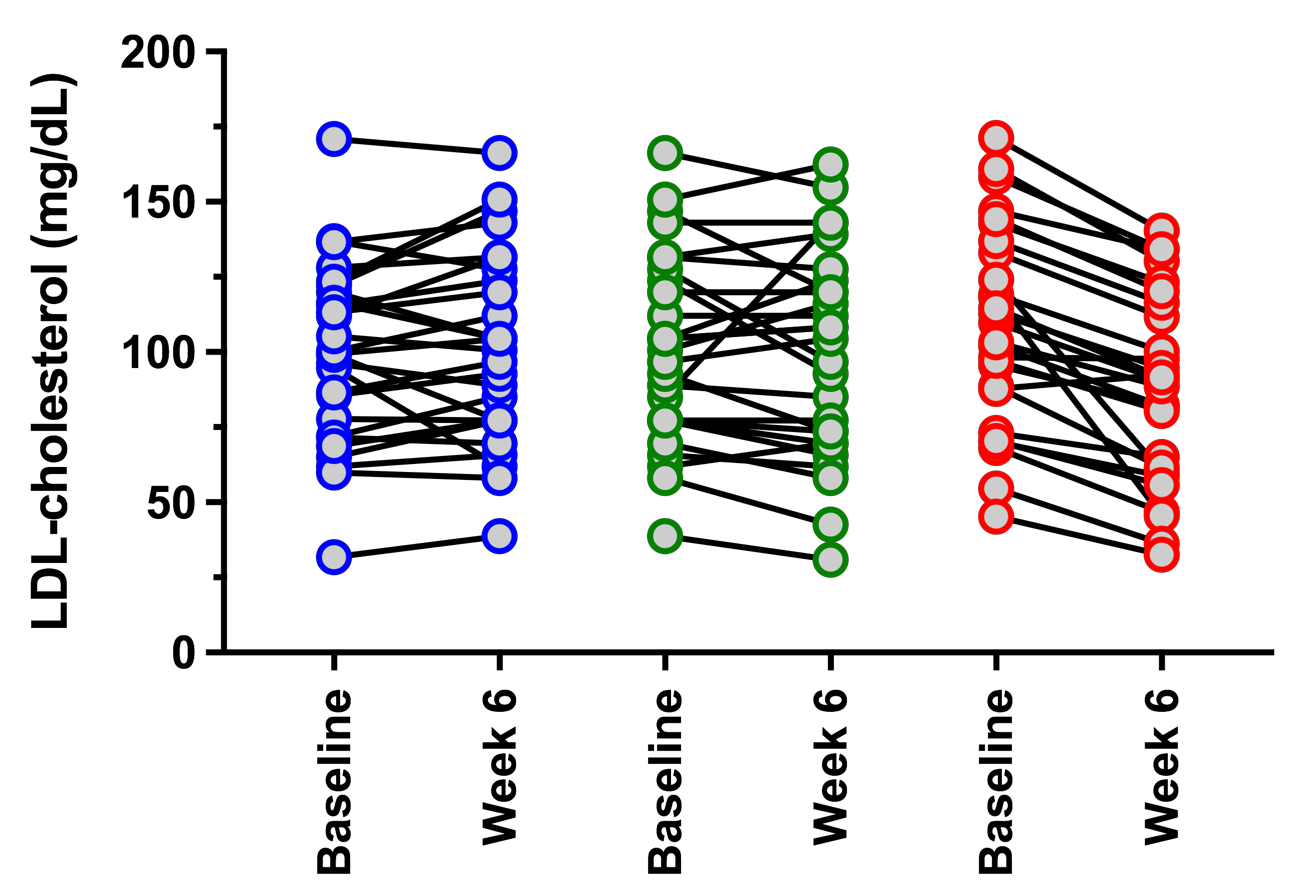

 Supplementary figure S4*. HDL cholesterol*


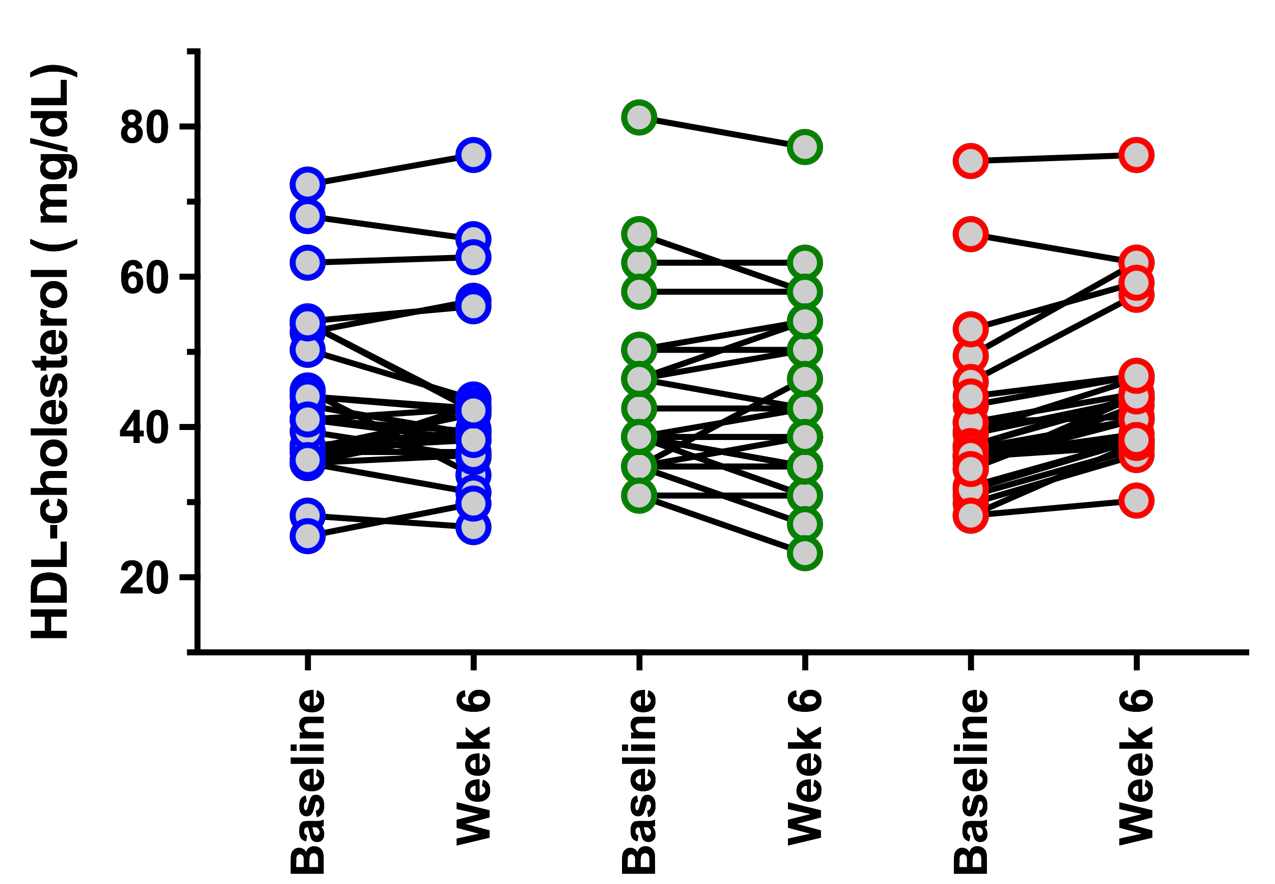


Supplementary figure S5*. Triglycerides*

**
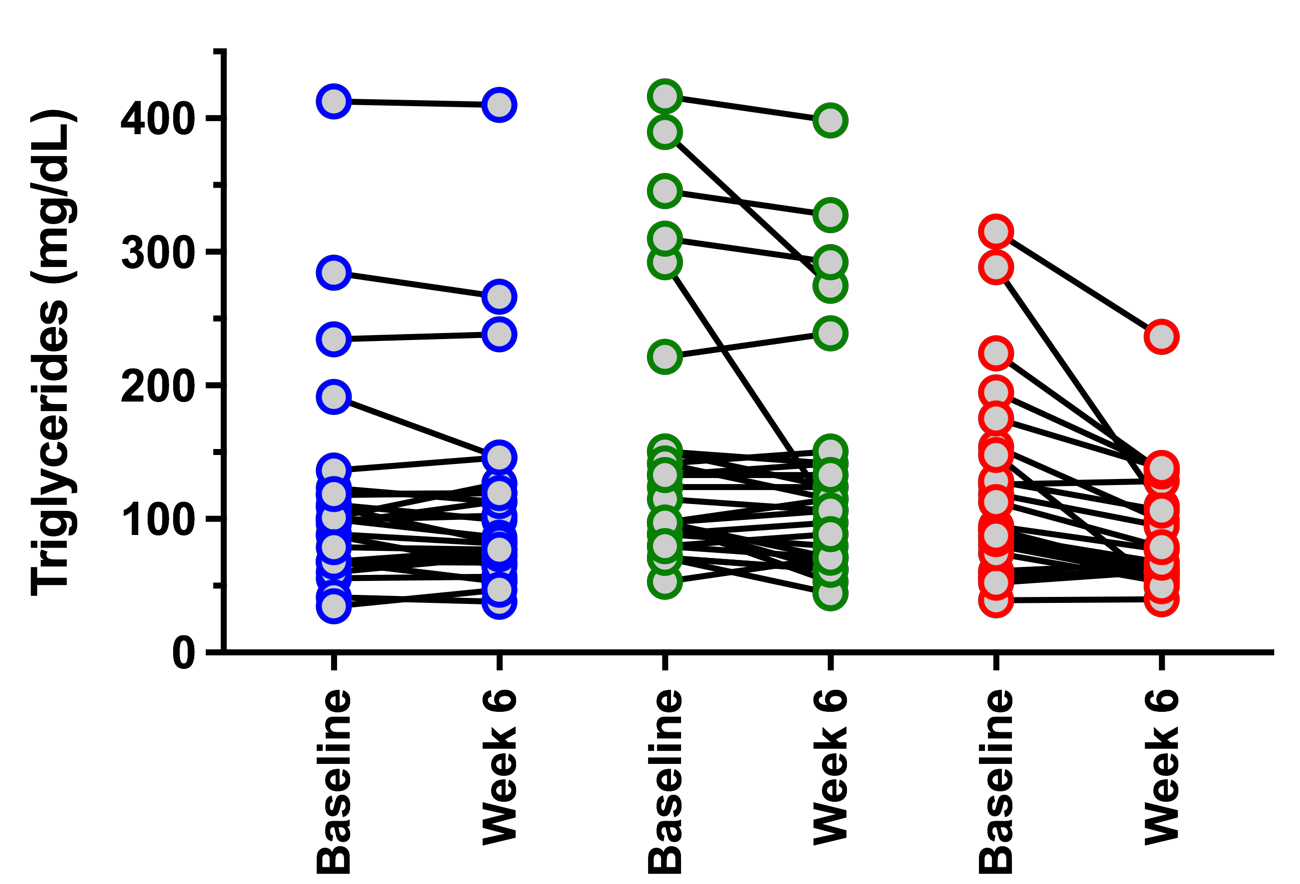
**

 Supplementary figure S6. *Lipoprotein(a)*


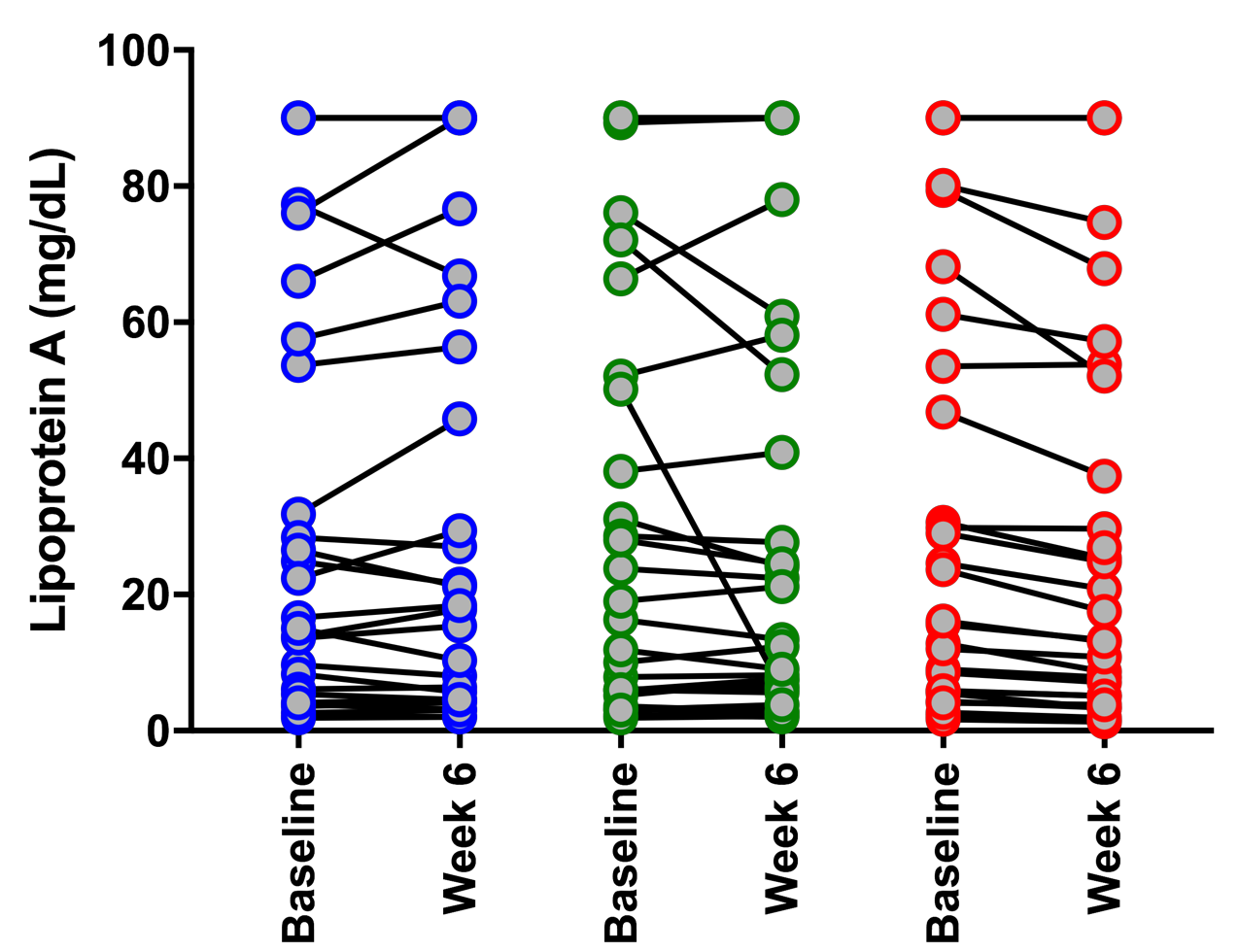


**7. Supplementary figure S7.** *Individual patient changes in PCSK9 for each treatment phase. ‘*Before and after’ plots of individual patient PCSK9 concentrations at baseline and week 6 for each treatment phase. Blue dots denote subjects on placebo; green dots denote subjects on nifedipine; red dots denote subjects on selective ET_A_ receptor antagonist. ET_A_ – endothelin-A; PCSK9 – proprotein convertase subtilisin/kexin type 9

**
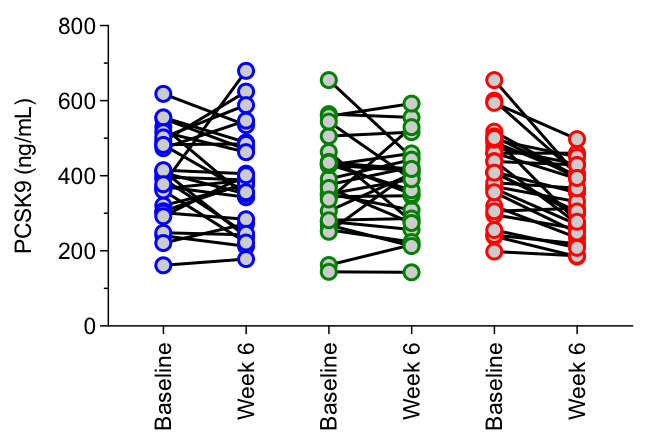
**

**8. Supplementary figure S8**: *Pattern of change in lipid profile with ET_A_ receptor antagonism compared to PCSK9 inhibition.* Bar chart showing representative change from baseline of lipid parameters following selective ET_A_ receptor antagonist (**A**) compared to PCSK9 inhibitors, evolocumab^2^ (**B)** and alirocumab^3^ (**C**). Bars represent percentage change from baseline at after 6 weeks treatment for ET_A_ receptor antagonist and after 12 weeks treatment for PCSK9 inhibitors. Chol – total cholesterol; LDL-C– low density lipoprotein cholesterol; HDL-C – high density lipoprotein cholesterol; TG – triglycerides; Lp(a) – lipoprotein(a)

**A.** ET_A_ antagonist


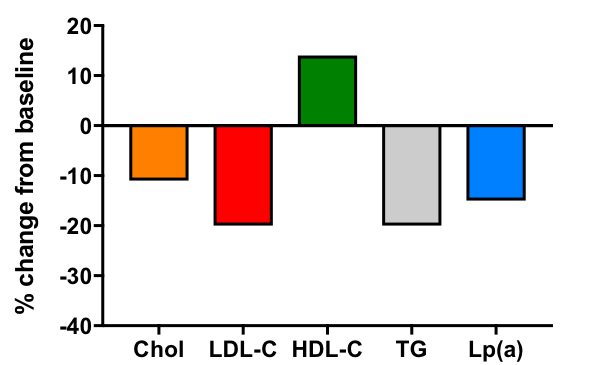


**B.** Evolocumab


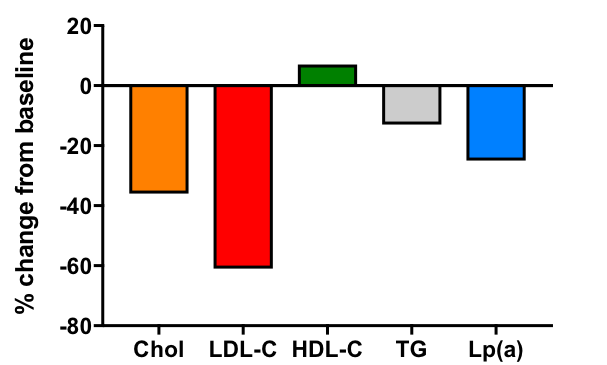


**C.** Alirocumab


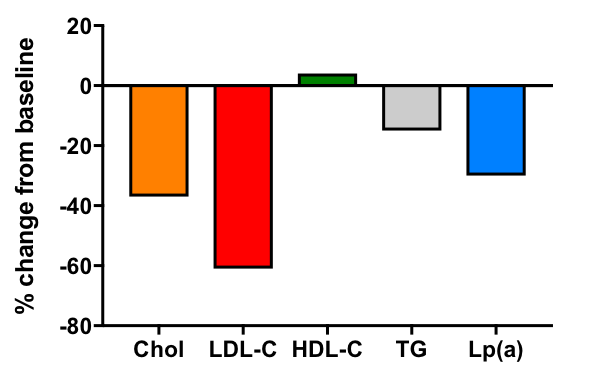

Supplement: Supplementary file 1 [file hyp-74-323-s001.docx]
