# Supplementary material for: Endothelin Receptor Antagonism Improves Lipid Profiles and Lowers PCSK9 (Proprotein Convertase Subtilisin/Kexin Type 9) in Patients With Chronic Kidney Disease
Source: Hypertension. 2019 Jul 10;74(2):323–30. doi: 10.1161/HYPERTENSIONAHA.119.12919 (PMC6635059; doi:10.1161/HYPERTENSIONAHA.119.12919)
Supplement: Supplementary file 2 [file hyp-74-323-s002.docx]

**Endothelin receptor antagonism improves lipid profiles & lowers PCSK9 in patients with chronic kidney disease**

Tariq E Farrah MD^* 1, 2^

Atul Anand MD^* 1^

Peter J Gallacher MD^1,2^

Robert Kimmitt MD^1^

Edwin Carter BSc^1^

James W Dear MD PhD^1^

Nicholas L Mills MD PhD^1^

David J Webb MD DSc^1^

Neeraj Dhaun MD PhD^1, 2^ ** Joint first authors*

^1^ University/British Heart Foundation Centre of Research Excellence, Centre of Cardiovascular Science, University of Edinburgh, Queen's Medical Research Institute, 47 Little France Crescent, Edinburgh, EH16 4TJ

^2^ Department of Renal Medicine, Royal Infirmary of Edinburgh, 51 Little France Crescent, Edinburgh, EH16 4SA

**Correspondence to**: Dr Neeraj Dhaun *(Bean)*

Centre for Cardiovascular Science

The Queen’s Medical Research Institute

47 Little France Crescent, Edinburgh.

EH16 4TJ

[bean.dhaun@ed.ac.uk](mailto:bean.dhaun@ed.ac.uk)

Tables: 2 Figures: 4

Pages: 28 Total word count: 5,731

**Running title**: Endothelin & lipids in CKD

**Acknowledgments**

TF is supported by a Clinical Research Training Fellowship from the *Medical Research Council* (MR/R017840/1). AA is supported by a Research Fellowship from *Chest Heart and Stroke Scotland* (15/A163). NLM is supported by the Butler Senior Research Fellowship (FS/16/14/32023) and Chair (CH/09/002) awards from the *British Heart Foundation.* ND is supported by a *BHF* Intermediate Clinical Research Fellowship (FS/13/30/29994).

***Abstract***

Dyslipidemia is common in chronic kidney disease (CKD). Despite statins, many patients fail to adequately lower lipids and remain at increased risk of cardiovascular disease. Selective endothelin-A (ET_A_) receptor antagonists reduce cardiovascular disease risk factors. Pre-clinical data suggest that ET_A_ antagonism has beneficial effects on circulating lipids. We assessed the effects of selective ET_A_ antagonism on circulating lipids and proprotein convertase subtilisin/kexin type 9 (PCSK9) in CKD.

This was a secondary analysis of a fully randomized, double-blind, three-phase, crossover study. Twenty-seven subjects with pre-dialysis CKD on optimal cardio- and renoprotective treatment, were randomly assigned to receive 6 weeks dosing with placebo, the selective ET_A_ receptor antagonist, sitaxentan, or long-acting nifedipine. We measured circulating lipids and PCSK9 at baseline and then after 3 and 6 weeks.

Baseline lipids and PCSK9 did not differ before each study phase. Whereas placebo and nifedipine had no effect on lipids, 6 weeks of ET_A_ antagonism significantly reduced total (-11±1%) and low-density lipoprotein associated (-20±3%) cholesterol, lipoprotein (a) (-16±2%) and triglycerides (-20±4%); high-density lipoprotein associated cholesterol increased (+14±2%), p <0.05 *vs.* baseline for all. Additionally, ET_A_ receptor antagonism, but neither placebo nor nifedipine, reduced circulating PCSK9 (-19±2%, p <0.001 *vs.* baseline, p <0.05 *vs.* nifedipine and placebo). These effects were independent of statin use and changes in blood pressure or proteinuria.

Selective ET_A_ antagonism improves lipid profiles in optimally-managed CKD patients, effects that may occur through a reduction in circulating PCSK9. ET_A_ receptor antagonism offers a potentially novel strategy to reduce cardiovascular disease risk in CKD.

**Funding**

This study received no external funding. The original study was funded by Pfizer Inc.

**Clinical Trial Registration:** [www.clinicaltrials.gov](http://www.clinicaltrials.gov)/[NCT00810732](https://clinicaltrials.gov/show/NCT00810732)

**Keywords:** statins, atherosclerosis, cardiovascular disease, endothelin

***Introduction***

Chronic kidney disease (CKD) is common and an important, independent risk factor for cardiovascular disease (CVD).^1^ This increased risk is partly explained by a high prevalence of traditional CVD risk factors, such as diabetes mellitus and hypertension.^2^ Dyslipidemia is also common in CKD and contributes to the development of accelerated atherosclerosis and CVD.^3^ HMG-CoA reductase inhibitors (statins) lower cholesterol, particularly low-density lipoprotein associated cholesterol (LDL-C) and have proven efficacy in the reduction of CVD risk in those with and without CKD.^4, 5^ However, despite the use of statins, many CKD patients continue to have elevated lipids.^6^ Furthermore, the side effects associated with these drugs can limit their use.^7^ Thus, novel therapies that might lower cholesterol both in patients established on statin treatment and in those intolerant of statins would be of major clinical value.

Proprotein convertase subtilisin/kexin type 9 (PCSK9) is a serine protease produced mainly in the liver and is an important regulator of tissue LDL receptor (LDL-R) expression and cholesterol homeostasis.^8^ In the circulation, PCSK9 binds to cell surface LDL-R promoting their lysosomal degradation, leading to a rise in circulating LDL-C. Inhibition of circulating PCSK9 using novel humanized monoclonal antibodies leads to important reductions in LDL-C in patients on and off statins.^9, 10^ Recent pre-clinical studies have shown that PCSK9 expression increases during systemic inflammation^11^ and with podocyte injury.^12^ Both are central features of CKD and contributed to by endothelin-1 (ET-1).^13^

ET-1 is the most potent endogenous vasoconstrictor and plays an important role in the development and progression of CKD.^13^ The major pathological effects of ET-1 are mediated *via* endothelin-A (ET_A_) receptors.^13^ Pre-clinical data using ET receptor antagonists have suggested beneficial effects on circulating lipids and atherosclerosis^14^ but subsequent clinical studies have produced conflicting results and none have explored potential mechanisms.^15-19^ Thus, we hypothesized that in a cohort of optimally-managed proteinuric CKD patients, selective ET_A_ receptor antagonism would lead to a reduction in circulating lipids and PCSK9.

***Methods***

Data relating to this study are available from the corresponding author upon reasonable request. To test the paradigm that selective ET_A_ antagonism can lower circulating lipids, we performed a secondary analysis of a single center, fully randomized, double-blind, three-phase, placebo-controlled crossover study in patients with varying degrees of proteinuric, pre-dialysis CKD, a population at significantly increased CVD risk. The full study protocol has been described previously and was carried out with subjects’ written consent and South East Scotland research ethics committee approval.^20^

*Patients and interventions*

Twenty-seven patients were enrolled and randomly assigned to receive the selective ET_A_ receptor antagonist, sitaxentan 100 mg once-daily, matched placebo or long-acting nifedipine 30 mg once-daily for 6 weeks in addition to their usual medications. Each phase was separated by a minimum 2-week washout period. We included patients aged 18-70 years of age with stable CKD stages 1-4 and proteinuria >300 mg/day. Patients with diabetes mellitus, nephrotic syndrome, significant cardiorespiratory comorbidity, peripheral vascular disease, liver disease and women of childbearing potential were excluded.

*Assessments*

Patients underwent assessments at baseline, week 3 and week 6 of each treatment phase which included blood sampling for biochemical analyses. Total cholesterol, LDL-C, high-density lipoprotein associated cholesterol (HDL-C), triglycerides, lipoprotein(a) (Lp(a)) and circulating PCSK9 were also assessed at these timepoints (**Supplementary figure S1)**.

*Sample collection and analysis*

Blood was collected into serum and EDTA tubes, immediately centrifuged at 2500*g* for 20 minutes at 4°C and stored at -80°C until analysis. Lipid parameters were analyzed from stored serum whilst PCSK9 was analyzed from stored plasma. Total cholesterol, LDL-C, HDL-C and triglycerides were measured by enzymatic colorimetric assays. For total cholesterol, the limit of detection and intra- and inter-assay coefficients of variation (CV) were 0.02 mmol/L, 0.8% and 1.3%, respectively. For LDL-C the limit of detection was 0.03 mmol/L with intra- and inter-assay CV of 1.4% and 2.2%, respectively. For HDL-C the limit of detection was 0.06 mmol/L with intra- and inter-assay CV of 1.7% and 5.0%, respectively. For triglycerides the limit of detection was 0.13 mmol/L with intra- and inter-assay CV of 0.8% and 1.7%, respectively. Lp(a) was quantified using a latex agglutination assay with a limit of detection of 0.83 mg/dL and intra- and inter-assay CV of 1.2% and 3.0%, respectively. PCSK9 was measured using an enzyme-linked immunosorbent assay (R&D systems) with a limit of detection of 0.096 ng/mL. Mean recovery of PCSK9 was 107% and cross-reactivity was 0% for LDL-R, PCSK1, 3 and 7. The intra-and inter-assay CV were 4.1% and 5.6%, respectively.

*Statistical analysis*

The original study was designed to detect significant changes in proteinuria using data from a prior study^21^ where an ET_A_ receptor antagonist was administered to 22 subjects in a crossover design leading to a reduction in proteinuria of approximately 0.7 g/day with an SD of 0.9 g/day. Using these data, the current study size had 80% power to detect such a difference at the 2-sided 5% significance level.

Baseline lipid levels were assessed by repeated measures one-way ANOVA with Tukey correction for multiple comparisons to assess carryover and period effect at the start of each treatment phase. A repeated measures 3-way ANOVA was used to assess for interactions between time, treatment and statin use for changes in lipids and PCSK9. Where Mauchly’s test indicated the sphericity assumption was not met, the Greenhouse-Geisser or Huynh-Feldt correction were used as appropriate. Changes from baseline to week 6 within treatment phases and between treatments phases at all time points were assessed by repeated measures 2-way ANOVA with Sidak’s and Tukey’s corrections for multiple comparisons, respectively. Predictors of change in PCSK9 concentrations were modelled by linear regression, adjusting for potential confounders. Data were analyzed with IBM^®^SPSS (version 24) and R (version 3.3.3). Significance was taken at the 5% level.

***Results***

Baseline patient characteristics are shown in **Table 1**. Baseline mean (±SEM) total cholesterol was 165±29 mg/dL, LDL-C 103±32 mg/dL, HDL-C 43±11 mg/dL mmol/L, triglycerides 143±113 mg/dL and Lp(a) 31±3 mg/dL. There were no differences in circulating lipids at the start of each study phase (**Table 2**). Eighteen (67%) patients were prescribed a statin whilst 24 (89%) patients were prescribed either an angiotensin converting enzyme inhibitor (ACEi) or angiotensin receptor blocker (ARB).

As previously reported,^20^ 6 weeks dosing with a selective ET_A_ antagonist and nifedipine reduced BP and arterial stiffness similarly but ET_A_ antagonism reduced proteinuria to a greater extent than nifedipine. Placebo did not affect these parameters. All patients completed all three phases and no serious adverse events were recorded during any treatment phase.^20^

In terms of effects on circulating lipids and PCSK9, there were no significant three-way interactions between time, treatment and statin use but significant interactions between time and treatment were present and analyzed further. (**Supplementary table S1**). Total cholesterol, LDL-C and HDL-C were unaffected by placebo or nifedipine. In contrast, 6 weeks of ET_A_ antagonism led to a fall in total cholesterol of 18±2 mg/dL, a reduction of ~11% (p <0.001 *vs.* baseline, p <0.001 *vs*. nifedipine and placebo at week 6, **Figure 1A**). The reduction in total cholesterol seen with ET_A_ antagonism comprised a fall in LDL-C of 21±3 mg/dL (~20% reduction, p <0.001 *vs.* baseline, p <0.001 *vs*. nifedipine and placebo at week 6, **Figure 1B**) and an increase in HDL-C of 5±1 mg/dL (~14% increase, p <0.001 *vs.* baseline, p <0.001 *vs*. nifedipine and placebo at week 6, **Figure 1C**). ET_A_ antagonism also led to reductions in triglycerides of 39±10 mg/dL (~20% fall, p <0.001 *vs*. baseline, p<0.05 *vs*. nifedipine and placebo at week 6, **Figure 1D**) and in Lp(a) of 3.2±0.8 mg/dL (~15% fall p <0.05 *vs.* baseline and placebo at week 6, **Figure 1E**). Detailed effects of ET_A_ antagonism compared to nifedipine and placebo are shown in **Supplementary tables S2-7** with individual patient responses shown in **Supplementary figures S2-S6**.

Mean (±SEM) baseline plasma PCSK9 concentration was 400±117 ng/mL and did not differ between the three phases of the study (**Table 2**). Following 6 weeks of ET_A_receptor antagonism, PCSK9 fell by -81±13 ng/mL (~20% reduction, p <0.001 *vs*. baseline, p <0.05 *vs*. nifedipine and placebo, **Figure 2** and **Supplementary figure S7**). Reductions in circulating PCSK9 during ET_A_antagonism were observed to occur with simultaneous reductions in total cholesterol, LDL-C and to a lesser degree with triglycerides, whilst also associating with increases in HDL-C (**Figure 3**). No relationship could be demonstrated for the nifedipine and placebo phases.

Linear regression modeling indicated that treatment with ET_A_antagonism was an independent predictor of change in PCSK9 concentration at 6 weeks after adjustment for age, sex, statin treatment and baseline CVD risk factors (adjusted β –73ng/mL, 95% CI -131 to -16, p =0.01, **Supplementary table S8**). In addition, selective ET_A_ antagonism remained an independent predictor of change in PCSK9 concentration at 6 weeks after adjustment for change in proteinuria, BP and pulse wave velocity (**Supplementary** **table S9**).

***Discussion***

Our study has several important findings. We provide new evidence of the broad beneficial effects of selective ET_A_ receptor antagonism on circulating lipids in patients with varying degrees of pre-dialysis CKD and residual proteinuria. Here, medium-term dosing with an ET_A_ antagonist resulted in clinically relevant reductions in total cholesterol, LDL-C and triglycerides with a significant increase in HDL-C. In addition, we saw a significant fall in Lp(a). Importantly, these improvements occurred in patients at high CVD risk, the majority of whom were already receiving recommended CVD prevention therapy with a statin and either an ACEi or ARB. Furthermore, we have shown that these lipid-lowering effects occurred with a concurrent reduction in circulating PCSK9. This previously unreported finding supports a link between the endothelin system and cholesterol homeostasis.

Lowering LDL-C has been shown to reduce the risk of major atherosclerotic events in a wide range of patients with CKD.^4^ However, despite current lipid-lowering treatments a number of patients fail to achieve target LDL-C.^6^ This is mirrored in our cohort of patients whose mean baseline fasting LDL-C was above the recommended 100 mg/dL despite a high rate of statin use.^6, 22^ Additionally, of the 18 patients (67%) receiving a statin, 11 were also prescribed the cholesterol-absorption inhibitor, ezetimibe. Thus, from a clinical perspective, it is important that the effects observed with ET_A_ receptor antagonism occurred on top of currently available therapies. Furthermore, suboptimal dosing and discontinuation of statins due to adverse effects remain significant challenges in clinical practice.^7^ For example, the risk of statin-induced myopathy is increased in patients with impaired renal function.^23^ Therefore, alternative agents, which might improve lipid profiles in this particularly high-risk group, whilst conferring broader CVD risk protection, would be of major clinical value.

Previous reports of the lipid-lowering effects of ET receptor antagonism are limited. Kowala *et al*. demonstrated that selective ET_A_ antagonism lowered total cholesterol, LDL-C and triglycerides in hyperlipidemic hamsters and this reduced aortic arch atherosclerosis.^14^ Data from clinical studies are conflicting with some noting an improvement in lipid profiles^15, 17^ whereas others have shown no effect.^18, 24^ Studies in CKD are limited to those with diabetic nephropathy. In a study using the mixed ET_A/B_ antagonist, avosentan, the authors reported a ~7% reduction in total cholesterol after 12 weeks dosing but found no effect on triglycerides; they did not report on LDL-C or HDL-C.^16^ Using the selective ET_A_ antagonist, atrasentan, de Zeeuw and colleagues showed similar effects after 12 weeks on total cholesterol, LDL-C and triglycerides to those seen in the current study but no change in HDL-C.^19^ Our data add to these studies and also demonstrate a beneficial effect on HDL-C and Lp(a), as well as suggesting a potential mechanism through a reduction of circulating PCSK9. Interestingly, these effects on lipids may relate to the relative ET_A_:ET_B_ receptor selectivity of the drug used: bosentan (non-selective) no effect on cholesterol;^18^ avosentan (ET_A_:ET_B_ ~300:1) ~7% reduction in cholesterol;^16^ atrasentan (ET_A_:ET_B_ ~1,200:1) ~9% reduction in cholesterol ^19^ and sitaxentan (ET_A_:ET_B_ ~6,500:1) ~11% reduction in cholesterol.^25^

Beyond its role in cholesterol homeostasis, a link between circulating PCSK9 concentration and CVD risk has emerged. Leander *et al.* recently showed in a middle-aged, non-CKD population that a greater circulating PCSK9 was associated with a higher risk of incident CVD, particularly thrombotic events. This remained the case even after adjusting for traditional CVD risk factors including LDL-C and statin use.^26^ The mean PCSK9 level in our study (~400 ng/mL) is comparable to the highest risk quartile identified by Leander and colleagues^26^ and is similar to that seen in other studies in CKD.^27^ The greater CVD risk associated with elevated PCSK9 concentrations may be due in part to its positive association with elevated Lp(a), a modified low-density lipoprotein species that can impair endogenous fibrinolysis.^28^ A recent meta-analysis of >29,000 patients found an independent and near-linear association between both elevated baseline and on-statin Lp(a) concentrations and CVD risk.^29^ This association holds true in CKD as shown in ~3,500 CKD patients from the Chronic Renal Insufficiency Cohort where a higher concentration of Lp(a) was independently associated with the incident myocardial infarction and death during ~7.5 years follow up.^30^ Targeting these two novel markers of CVD risk using conventional treatments is challenging as statins increase circulating PCSK9^31^ and are ineffective at lowering Lp(a).^32^ Our data suggest that ET_A_ receptor antagonism reduces both.

Inhibition of circulating PCSK9 using novel humanized monoclonal antibodies results in marked reductions in LDL-C, and uniquely Lp(a), in clinical trials of patients both on and off statins.^9, 10^ These agents bind to circulating PCSK9, lowering plasma levels by ~90%.^33^ However, their widespread use is limited by cost; the current list price of evolocumab is more than $14,000 a year per patient.^34^ In our study, ET_A_ receptor antagonism reduced circulating PCSK9 by ~20%, which was associated with significant improvements in lipids. The pattern of improvement in lipid profiles with ET_A_ receptor antagonism, particularly the significant reductions in Lp(a) (~16%), is strikingly similar to that observed in clinical trials of PCSK9 inhibitors^9, 10^ (**Supplementary figure S8**) suggesting that these are secondary to a reduction in circulating PCSK9.

Our observed reduction in PCSK9 during ET_A_ antagonism occurred independently of the major recognized vascular and renal effects of this drug class, namely reductions in BP, arterial stiffness and proteinuria. This suggests novel and specific links between the endothelin system and PCSK9 as proposed in **Figure 4**. Systemic inflammation, podocyte injury and proteinuria are features of CKD and in which ET_A_ receptor activation plays a key role.^13^ All three have also been shown to increase tissue and circulating PCSK9 expression in mice.^11,12^ However, there was inconsistent evidence in these studies of a concurrent upregulation of sterol regulatory element binding protein-2 (SREBP-2) and hepatic nuclear factor 1-alpha (HNF1α), the principle promoters of PCSK9 expression. Induction of endoplasmic reticulum stress in murine renal proximal tubular cells has been shown to increase SREBP-2 expression with subsequent lipid accumulation and apoptosis.^35^ In a separate study, using the same model, selective ET_A_ antagonism reduced endoplasmic reticulum stress and apoptosis of proximal tubular cells.^36^ These studies did not examine PCSK9 expression but recent work suggests that increased renal PCSK9 expression after podocyte injury/ablation is also localized to proximal tubular cells.^12^

Other pre-clinical data suggest an important role for insulin in reducing hepatic PCSK9 expression by preventing nuclear translocation of HNF1α.^37^ ET-1 has been to shown to promote hepatic insulin resistance^38^ which can be restored by ET_A_ receptor antagonism in Zucker fatty rats ^39^ and in man^40^ with a subsequent improvement in glucose metabolism. Finally, inflammation may act as an important shared pathway as vascular smooth muscle cells, the predominant site of ET_A_ receptor expression, have recently been shown to express PCSK9^41^ which can be upregulated by inflammatory stimuli *in vitro*.^42^

The collected published data link the endothelin system, PCSK9 and lipid homeostasis (**Figure 4**) and provide a plausible mechanistic basis for our clinical observations that should be explored further in future studies. We recognize the small size of our study as well as its medium-term duration and observational nature and whilst our data are secondary analyses, they originate from a well-designed, fully randomized, placebo-controlled clinical trial with no measurable evidence of carryover or period effects. We acknowledge that whilst the 2-week washout period between phases was designed to ensure adequate drug elimination, persisting effects on cholesterol metabolism cannot be fully excluded, although our detailed analyses show no sign of this. We demonstrate clear, consistent benefits on circulating lipids with ET_A_ antagonism and provide novel insight into links between the endothelin system and cholesterol homeostasis in kidney disease.

***Perspectives***

Medium-term selective ET_A_ antagonism improves lipid profiles in optimally-managed CKD patients. Our data suggest that the lipid lowering effects of ET_A_ antagonism may be achieved through a reduction in circulating PCSK9. Alongside recognized reductions in BP, proteinuria and arterial stiffness, ET_A_ receptor antagonism offers a novel strategy to reduce CVD risk in patients with CKD. Current larger clinical trials of selective ET_A_ antagonism alone^43^ or in combination with angiotensin II receptor blockade^43, 44^ in patients with proteinuric CKD should help confirm the current observations.

***Author contributions***

TF and AA drafted the manuscript, performed statistical analysis and critically revised the manuscript. PJG performed statistical analysis and critically revised the manuscript. RK critically revised the manuscript. EC performed biochemical assays. JWD, NLM and DJW critically revised the manuscript. ND conceived the study, carried out the primary study and critically revised the manuscript. All authors approved the final version.

***Acknowledgments***

*None*

***Sources of funding***

TF is supported by a Clinical Research Training Fellowship from the *Medical Research Council* (MR/R017840/1). AA is supported by a Research Fellowship from *Chest Heart and Stroke Scotland* (15/A163). NLM is supported by the Butler Senior Research Fellowship (FS/16/14/32023) and Chair (CH/09/002) awards from the *British Heart Foundation.* ND is supported by a *BHF* Intermediate Clinical Research Fellowship (FS/13/30/29994).

***Financial disclosures***

ND has acted as a consultant for Retrophin Inc. All other authors have no financial disclosures.

financial disclosures.

**References**

1. James MT, Hemmelgarn BR, Tonelli M: Early recognition and prevention of chronic kidney disease. *Lancet,* 375: 1296-1309, 2010.

2. Gansevoort RT, Correa-Rotter R, Hemmelgarn BR, Jafar TH, Heerspink HJ, Mann JF, Matsushita K, Wen CP: Chronic kidney disease and cardiovascular risk: epidemiology, mechanisms, and prevention. *Lancet,* 382: 339-352, 2013.

3. Kwan BC, Kronenberg F, Beddhu S, Cheung AK: Lipoprotein metabolism and lipid management in chronic kidney disease. *J Am Soc Nephrol,* 18: 1246-1261, 2007.

4. Baigent C, Landray MJ, Reith C, Emberson J, Wheeler DC, Tomson C*, et al.*: The effects of lowering LDL cholesterol with simvastatin plus ezetimibe in patients with chronic kidney disease (Study of Heart and Renal Protection): a randomised placebo-controlled trial. *Lancet,* 377: 2181-2192, 2011.

5. Silverman MG, Ference BA, Im K, et al.: Association between lowering LDL-C and cardiovascular risk reduction among different therapeutic interventions: A systematic review and meta-analysis. *JAMA,* 316: 1289-1297, 2016.

6. Foster MC, Rawlings AM, Marrett E, Neff D, Grams ME, Kasiske BL, Willis K, Inker LA, Coresh J, Selvin E: Potential effects of reclassifying CKD as a coronary heart disease risk equivalent in the US population. *Am J Kidney Dis,* 63: 753-760, 2014.

7. Zhang H, Plutzky J, Skentzos S, Morrison F, Mar P, Shubina M, Turchin A: Discontinuation of statins in routine care settings: a cohort study. *Ann Intern Med,* 158: 526-534, 2013.

8. Zhang DW, Lagace TA, Garuti R, Zhao Z, McDonald M, Horton JD, Cohen JC, Hobbs HH: Binding of proprotein convertase subtilisin/kexin type 9 to epidermal growth factor-like repeat A of low density lipoprotein receptor decreases receptor recycling and increases degradation. *J Biol Chem,* 282: 18602-18612, 2007.

9. Sabatine MS, Giugliano RP, Wiviott SD, Raal FJ, Blom DJ, Robinson J, Ballantyne CM, Somaratne R, Legg J, Wasserman SM, Scott R, Koren MJ, Stein EA, Open-Label Study of Long-Term Evaluation against LDLCI: Efficacy and safety of evolocumab in reducing lipids and cardiovascular events. *N Engl J Med,* 372: 1500-1509, 2015.

10. Robinson JG, Farnier M, Krempf M, Bergeron J, Luc G, Averna M*, et al.*: Efficacy and safety of alirocumab in reducing lipids and cardiovascular events. *N Engl J Med,* 372: 1489-1499, 2015.

11. Feingold KR, Moser AH, Shigenaga JK, Patzek SM, Grunfeld C: Inflammation stimulates the expression of PCSK9. *Biochem Biophys Res Commun,* 374: 341-344, 2008.

12. Haas ME, Levenson AE, Sun X, Liao WH, Rutkowski JM, de Ferranti SD, Schumacher VA, Scherer PE, Salant DJ, Biddinger SB: The role of proprotein convertase subtilisin/kexin type 9 in nephrotic syndrome-associated hypercholesterolemia. *Circulation,* 134: 61-72, 2016.

13. Dhaun N, Goddard J, Webb DJ: The endothelin system and its antagonism in chronic kidney disease. *J Am Soc Nephrol,* 17: 943-955, 2006.

14. Kowala MC, Rose PM, Stein PD, Goller N, Recce R, Beyer S, Valentine M, Barton D, Durham SK: Selective blockade of the endothelin subtype A receptor decreases early atherosclerosis in hamsters fed cholesterol. *Am J Pathol,* 146: 819-826, 1995.

15. Raichlin E, Prasad A, Mathew V, Kent B, Holmes DR, Jr., Pumper GM, Nelson RE, Lerman LO, Lerman A: Efficacy and safety of atrasentan in patients with cardiovascular risk and early atherosclerosis. *Hypertension,* 52: 522-528, 2008.

16. Wenzel RR, Littke T, Kuranoff S, Jurgens C, Bruck H, Ritz E, Philipp T, Mitchell A: Avosentan reduces albumin excretion in diabetics with macroalbuminuria. *J Am Soc Nephrol,* 20: 655-664, 2009.

17. Reriani M, Raichlin E, Prasad A, Mathew V, Pumper GM, Nelson RE, Lennon R, Rihal C, Lerman LO, Lerman A: Long-term administration of endothelin receptor antagonist improves coronary endothelial function in patients with early atherosclerosis. *Circulation,* 122: 958-966, 2010.

18. Rafnsson A, Bohm F, Settergren M, Gonon A, Brismar K, Pernow J: The endothelin receptor antagonist bosentan improves peripheral endothelial function in patients with type 2 diabetes mellitus and microalbuminuria: a randomised trial. *Diabetologia,* 55: 600-607, 2012.

19. de Zeeuw D, Coll B, Andress D, Brennan JJ, Tang H, Houser M*, et al.*: The endothelin antagonist atrasentan lowers residual albuminuria in patients with type 2 diabetic nephropathy. *J Am Soc Nephrol,* 25: 1083-1093, 2014.

20. Dhaun N, MacIntyre IM, Kerr D, Melville V, Johnston NR, Haughie S, Goddard J, Webb DJ: Selective endothelin-A receptor antagonism reduces proteinuria, blood pressure, and arterial stiffness in chronic proteinuric kidney disease. *Hypertension,* 57: 772-779, 2011.

21. Dhaun N, Macintyre IM, Melville V, Lilitkarntakul P, Johnston NR, Goddard J, Webb DJ: Blood pressure-independent reduction in proteinuria and arterial stiffness after acute endothelin-a receptor antagonism in chronic kidney disease. *Hypertension,* 54: 113-119, 2009.

22. Writing C, Lloyd-Jones DM, Morris PB, Ballantyne CM, Birtcher KK, Daly DD, Jr., DePalma SM, Minissian MB, Orringer CE, Smith SC, Jr.: 2016 ACC Expert Consensus Decision Pathway on the Role of Non-Statin Therapies for LDL-Cholesterol Lowering in the Management of Atherosclerotic Cardiovascular Disease Risk: A Report of the American College of Cardiology Task Force on Clinical Expert Consensus Documents. *J Am Coll Cardiol,* 68: 92-125, 2016.

23. The SEARCH Collaborative Group: SLCO1B1 variants and statin-induced myopathy — a genomewide study. *N Engl J Med,* 359: 789-799, 2008.

24. Schreuder TH, Duncker DJ, Hopman MT, Thijssen DH: Randomized controlled trial using bosentan to enhance the impact of exercise training in subjects with type 2 diabetes mellitus. *Exp Physiol,* 99: 1538-1547, 2014.

25. Kohan DE, Pollock DM: Endothelin antagonists for diabetic and non-diabetic chronic kidney disease. *Br J Clin Pharmacol,* 76: 573-579, 2013.

26. Leander K, Malarstig A, Van't Hooft FM, Hyde C, Hellenius ML, Troutt JS, Konrad RJ, Ohrvik J, Hamsten A, de Faire U: Circulating proprotein convertase subtilisin/kexin type 9 (PCSK9) predicts future risk of cardiovascular events independently of established risk factors. *Circulation,* 133: 1230-1239, 2016.

27. Rogacev KS, Heine GH, Silbernagel G, Kleber ME, Seiler S, Emrich I, Lennartz S, Werner C, Zawada AM, Fliser D, Böhm M, März W, Scharnagl H, Laufs U: PCSK9 plasma concentrations are independent of GFR and do not predict cardiovascular events in patients with decreased GFR. *PLoS One,* 11: e0146920, 2016.

28. Tavori H, Christian D, Minnier J, Plubell D, Shapiro MD, Yeang C, Giunzioni I, Croyal M, Duell PB, Lambert G, Tsimikas S, Fazio S: PCSK9 association With lipoprotein(a). *Circ Res,* 119: 29, 2016.

29. Willeit P, Ridker PM, Nestel PJ, Simes J, Tonkin AM, Pedersen TR*, et al.*: Baseline and on-statin treatment lipoprotein(a) levels for prediction of cardiovascular events: individual patient-data meta-analysis of statin outcome trials. *Lancet,* 392: 1311-1320, 2018.

30. Bajaj A, Damrauer SM, Anderson AH, Xie D, Budoff MJ, Go AS*, et al.*: Lipoprotein(a) and risk of myocardial infarction and death in chronic kidney disease: Findings from the CRIC study (Chronic Renal Insufficiency Cohort). *Arterioscler Thromb Vasc Biol,* 37: 1971-1978, 2017.

31. Careskey HE, Davis RA, Alborn WE, Troutt JS, Cao G, Konrad RJ: Atorvastatin increases human serum levels of proprotein convertase subtilisin/kexin type 9. *J Lipid Res,* 49: 394-398, 2008.

32. Kostner GM, Gavish D, Leopold B, Bolzano K, Weintraub MS, Breslow JL: HMG CoA reductase inhibitors lower LDL cholesterol without reducing Lp(a) levels. *Circulation,* 80: 1313, 1989.

33. Dias CS, Shaywitz AJ, Wasserman SM, Smith BP, Gao B, Stolman DS*, et al.*: Effects of AMG 145 on low-density lipoprotein cholesterol levels: results from 2 randomized, double-blind, placebo-controlled, ascending-dose phase 1 studies in healthy volunteers and hypercholesterolemic subjects on statins. *J Am Coll Cardiol,* 60: 1888-1898, 2012.

34. Fonarow GC, Keech AC, Pedersen TR, et al.: Cost-effectiveness of evolocumab therapy for reducing cardiovascular events in patients with atherosclerotic cardiovascular disease. *JAMA Cardiology,* 2: 1069-1078, 2017.

35. Lhoták Š, Sood S, Brimble E, Carlisle RE, Colgan SM, Mazzetti A, Dickhout JG, Ingram AJ, Austin RC: ER stress contributes to renal proximal tubule injury by increasing SREBP-2-mediated lipid accumulation and apoptotic cell death. *Am J Physiol,* 303: F266, 2012.

36. De Miguel C, Hamrick WC, Hobbs JL, Pollock DM, Carmines PK, Pollock JS: Endothelin receptor-specific control of endoplasmic reticulum stress and apoptosis in the kidney. *Sci Rep,* 7: 43152, 2017.

37. Ai D, Chen C, Han S, Ganda A, Murphy AJ, Haeusler R, Thorp E, Accili D, Horton JD, Tall AR: Regulation of hepatic LDL receptors by mTORC1 and PCSK9 in mice. *J Clin Invest,* 122: 1262-1270, 2012.

38. Ottosson-Seeberger A, Lundberg JM, Alvestrand A, Ahlborg G: Exogenous endothelin-1 causes peripheral insulin resistance in healthy humans. *Acta Physiol Scand,* 161: 211-220, 1997.

39. Berthiaume N, Carlson CJ, Rondinone CM, Zinker BA: Endothelin antagonism improves hepatic insulin sensitivity associated with insulin signaling in Zucker fatty rats. *Metabolism,* 54: 1515-1523, 2005.

40. Ahlborg G, Lindstrom J: Insulin sensitivity and big ET-1 conversion to ET-1 after ETA- or ETB-receptor blockade in humans. *J Appl Physiol (1985),* 93: 2112-2121, 2002.

41. Ferri N, Tibolla G, Pirillo A, Cipollone F, Mezzetti A, Pacia S, Corsini A, Catapano AL: Proprotein convertase subtilisin kexin type 9 (PCSK9) secreted by cultured smooth muscle cells reduces macrophages LDLR levels. *Atherosclerosis,* 220: 381-386.

42. Stawowy P, Meyborg H, Bezhaeva T, Fritzsche J, Molnar S, Urban D, Fleck E: Expression and regulation of proprotein convertase subtilisin/kexin type 9 in vascular smooth muscle cells. *Eur Heart J,* 34: P4166-P4166, 2013.

43. US National Library of Medicine. A study of the effect and safety of sparsentan in the treatment of patients with IgA nephropathy (PROTECT). <https://clinicaltrials.gov/ct2/show/NCT03762850>. Accessed:23rd April 2019

44. US National Library of Medicine. Randomized, double-blind, safety and efficacy study of RE-021 (sparsentan) in focal segmental glomerulosclerosis (DUET). <https://clinicaltrials.gov/ct2/show/NCT01613118>. Accessed:31st Jan 2019

**Novelty & Significance**

*What is new?*

- In patients with chronic kidney disease, treatment with a selective ET_A_ antagonist led to clinically-relevant reductions in circulating lipids.
- Interestingly, these lipid-lowering effects occurred alongside a reduction in circulating PCSK9.

*What is relevant?*

- Patients with chronic kidney disease are at high cardiovascular risk. Dyslipidemia is common and despite the use of statins, many of these patients continue to have elevated lipids.
- The beneficial effects seen here were in patients at high cardiovascular risk, the majority of whom were already receiving recommended cardiovascular disease prevention therapy.

*Summary*

- In patients with pre-dialysis chronic kidney disease, selective ET_A_ receptor antagonism reduces circulating lipids. These effects are seen on top of statin therapy and may be mediated through a reduction in PCSK9. These previously unreported findings support a link between the endothelin system and cholesterol homeostasis.

**Figure legends**

**Figure 1.** *Changes in lipid profiles*

Bar chart of mean change from baseline of total cholesterol (**A**), LDL-C (**B**), HDL-C (**C**), triglycerides (**D**) and Lp(a) (**E**) after week 3 and week 6 of dosing with placebo (blue bars), nifedipine (green bars) and selective ET_A_ receptor antagonism (red bars). ***p <0.001 for change at week 6 *vs.* baseline; ^•••^p <0.001 for change at timepoint *vs.* placebo and nifedipine ^•^p<0.05 for change at timepoint *vs.* placebo or nifedipine. Analysis by ANOVA. Error bars are standard error of mean. Conversion factors for units: cholesterol, LDL-C, HDL-C in mg/dL to mmol/L, x 0.02586; triglycerides in mg/dL to mmol/L, x 0.01129.

**Figure 2.** *Change in circulating PCSK9*

Bar chart of mean change in plasma PCSK9 from baseline after week 3 and week 6 of dosing with placebo (blue bars), nifedipine (green bars) and selective ET_A_ receptor antagonist (red bars). ***p <0.001 for selective ET_A_ receptor antagonist at week 6 *vs.* baseline; analysis by paired *t*-tests. ^•^p <0.05 for change at timepoint *vs.* placebo and nifedipine. Analysis by ANOVA. Error bars are standard error of mean.

**Figure 3.** *Change in lipids and circulating PCSK9*

Scatter plots of individual percentage changes from baseline in total cholesterol (**A**), LDL-C (**B**), HDL-C (**C**) and triglycerides (**D**) after 6 weeks of treatment *versus* individual percentage change in plasma PCSK9. Blue dots denote subjects receiving placebo; green dots denote subjects receiving nifedipine, red dots denote subjects receiving selective ET_A_ receptor antagonism.

**Figure 4.** *Proposed pathways linking the endothelin system, PCSK9 expression and cholesterol in chronic kidney disease.*

The liver is the major site of PCSK9 expression with hepatic nuclear factor 1α (HNF1α) and sterol regulatory element binding protein-2 (SREBP-2) its principle promoters.^4^ Animals studies have shown that insulin binding to hepatocytes prevents nuclear translocation of HNF1α thus reducing PCSK9 transcription.^5^ Endothelin-1 (ET-1) impairs hepatocyte insulin sensitivity^6^ which can be ameliorated by selective ET_A_ receptor antagonism^7, 8^ and so may restore the inhibitory effect of insulin on HNF1α-mediated PCSK9 transcription in hepatocytes. Whether ET-1 has direct effects on HNF1α or SREBP-2 in hepatocytes is unknown. Systemic inflammation can increase both hepatic and renal PCSK9 expression with a concurrent reduction in LDL receptor (LDL-R) expression^9^ and a rise in low density lipoprotein-associated cholesterol (LDL-C). In the vasculature, ET-1 has pro-inflammatory effects mediated predominantly through ET_A_ receptor activation.^10^ In the kidney, podocyte damage is associated with increased circulating and renal PCSK9 expression, notably localized to proximal tubular cells in murine models.^11^ The relevance of renal PCSK9 expression to the circulating PCSK9 pool and lipids needs further clarification. Interestingly, ER stress leads to an upregulation of SREBP-2 in renal proximal tubular cells with subsequent apoptosis^12^ but effects on PCSK9 expression here are unexplored. However selective ET_A_ antagonism has been shown to ameliorate podocyte injury^13^ and proximal tubule ER stress^14^ suggesting a potential role in renal PCSK9 expression. PCSK9 – proprotein convertase subtilisin kexin type 9.

**Table 1.** *Baseline study characteristics*. Values are mean ± standard error of 3 baseline treatment periods unless stated.

| **Parameter** | |
| --- | --- |
| ***Demographic*** |  |
| Age, years | 48±12 |
| Male (%) | 23 (85) |
| ***Clinical*** |  |
| 24hr average systolic BP, mmHg | 125±12 |
| 24hr average diastolic BP, mmHg | 78±7 |
| Pulse wave velocity, m/s | 7.9±0.3 |
| ***Biochemistry*** |  |
| Creatinine, mg/dL | 1.73±0.85 |
| eGFR, ml/min/1.73m^2^ | 54±26 |
| CKD stage by eGFR, n (%) |  |
| 1 | 5 (19) |
| 2 | 6 (22) |
| 3 | 11 (40) |
| 4 | 5 (19) |
| 5 | 0 |
| Urine protein-to-creatinine ratio, mg/mmol | 156±29 |
| Total cholesterol, mg/dL | 165±29 |
| LDL-C, mg/dL | 103±32 |
| HDL-C, mg/dL | 43±11 |
| Triglycerides, mg/dL | 143±113 |
| Lp(a), mg/dL | 30±3 |
| PCSK9, ng/mL | 400±117 |
| ***Medications, n (%)*** |  |
| Statin | 18 (67) |
| ACEi | 18 (67) |
| ARB | 11 (41) |
| α-blocker | 6 (22) |
| β-blocker | 8 (30) |
| Calcium channel blocker | 3 (11) |
| Diuretic | 2 (7) |

BP –blood pressure; eGFR – estimated glomerular filtration rate by Modified Diet in Renal Disease equation; LDL-C – low density lipoprotein associated cholesterol; HDL-C – high density lipoprotein associated cholesterol; Lp(a) – lipoprotein (a); PCSK9 – proprotein convertase subtilisin/kexin type 9; ACEi – angiotensin converting enzyme inhibitor; ARB – angiotensin receptor blocker. CKD – chronic kidney disease. Conversion factors for units: serum creatinine in mg/dL to umol/L, x 88.4; cholesterol, LDL-C, HDL-C in mg/dL to mmol/L, x 0.02586; triglycerides in mg/dL to mmol/L, x 0.01129.

**Table 2.** *Baseline lipid profiles for each study phase*. Values are pre-dosing mean ± standard error, mg/dL unless stated. Analysis by ANOVA. LDL-C – low density lipoprotein associated cholesterol; HDL-C – high density lipoprotein associated cholesterol; Lp(a) – lipoprotein (a); PCSK9 – proprotein convertase subtilisin/kexin type 9.

| **Parameter** | **Placebo** | **Nifedipine** | **ET_A_ antagonist** | ***p value*** |
| --- | --- | --- | --- | --- |
| **Total cholesterol** | 164±6 | 167±6 | 164±6 | 0.9 |
| **LDL-C** | 99±6 | 101±6 | 109±6 | 0.6 |
| **HDL-C** | 44±2 | 43±2 | 40±2 | 0.6 |
| **Triglycerides** | 116±16 | 153±20 | 137±23 | 0.8 |
| **Lp(a)** | 30 ± 6 | 31 ± 6 | 30 ± 6 | 0.9 |
| **PCSK9 (ng/mL)** | 400 ± 22 | 386 ± 24 | 414 ± 22 | 0.9 |

LDL-C – low density lipoprotein associated cholesterol; HDL-C – high density lipoprotein associated cholesterol; Lp(a) – lipoprotein (a); PCSK9 – proprotein convertase subtilisin/kexin type 9. Conversion factors for units: cholesterol, LDL-C, HDL-C in mg/dL to mmol/L, x 0.02586; triglycerides in mg/dL to mmol/L, x 0.01129.
