# Supplementary figures and images for: Endothelin Receptor Antagonism Improves Lipid Profiles and Lowers PCSK9 (Proprotein Convertase Subtilisin/Kexin Type 9) in Patients With Chronic Kidney Disease
Source: Hypertension. 2019 Jul 10;74(2):323–30. doi: 10.1161/HYPERTENSIONAHA.119.12919 (PMC6635059; doi:10.1161/HYPERTENSIONAHA.119.12919)

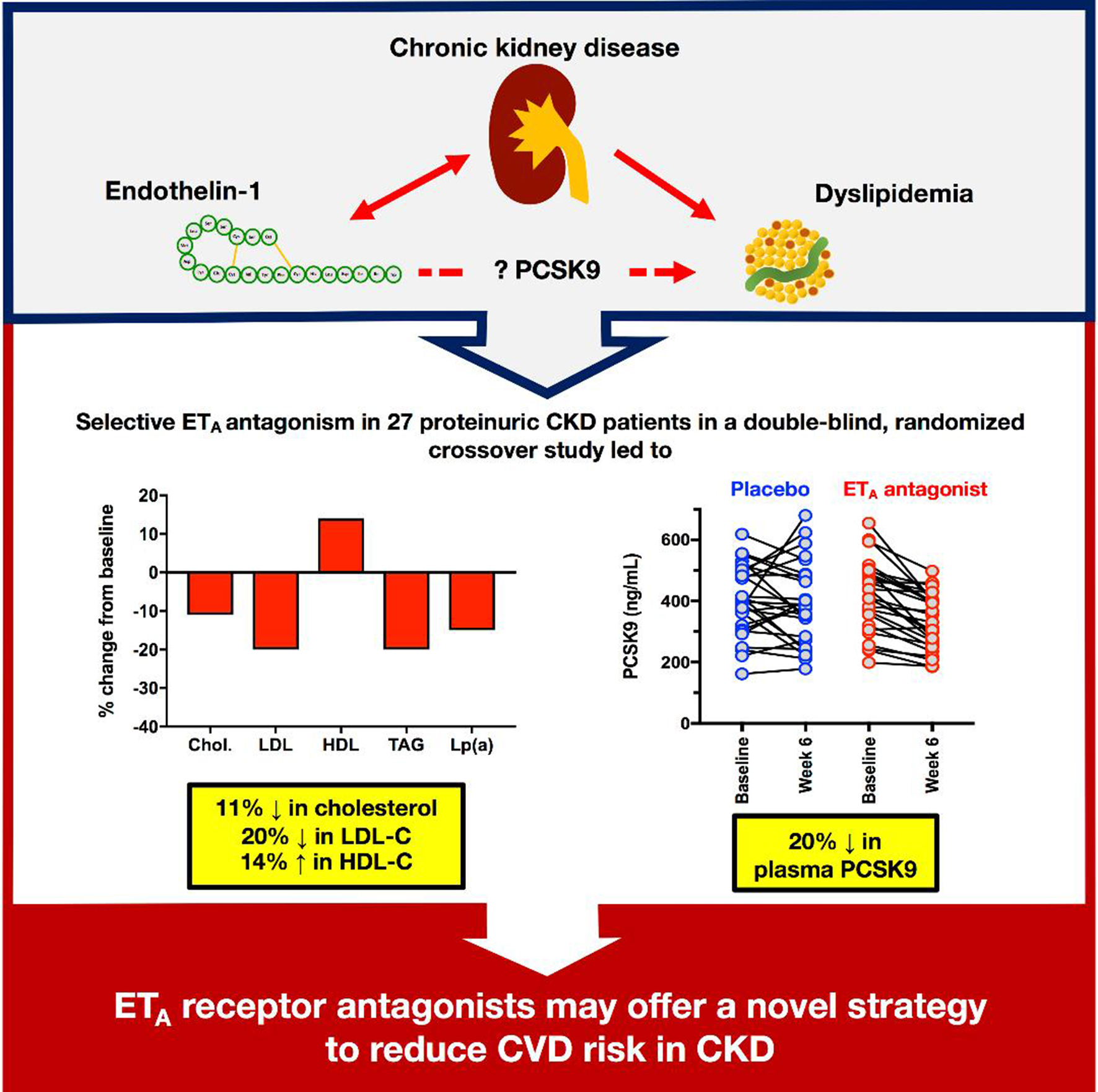

Supplement: Supplementary file 4 [file hyp-74-323-s004.jpg]
